# Role of mitochondria in physiological activities, diseases, and therapy

**DOI:** 10.1186/s43556-025-00284-5

**Published:** 2025-06-19

**Authors:** Lilin Wang, Xiaoting Zhou, Tianqi Lu

**Affiliations:** 1 Sichuan Engineering Research Center for Biomimetic Synthesis of Natural Drugs, School of Life Science and Engineering, Southwest Jiaotong University, Chengdu, Sichuan China; 2 Obesity and Metabolism Medicine-Engineering Integration Laboratory, Department of General Surgery, The Third People’s Hospital of Chengdu, Chengdu, China; 3 Center of Gastrointestinal and Minimally Invasive Surgery, Department of General Surgery, Affiliated Hospital of Southwest Jiaotong University, The Third People’s Hospital of Chengdu, Chengdu, China; 4 Medical Research Center, Affiliated Hospital of Southwest Jiaotong University, The Third People’s Hospital of Chengdu, Chengdu, Sichuan China; 5 Department of Gynecology and Obstetrics, Development and Related Disease of Women and Children Key Laboratory of Sichuan Province, Key Laboratory of Birth Defects and Related Diseases of Women and Children, Ministry of Education, West China Second Hospital, Sichuan University, Chengdu, 610041 People’s Republic of China

**Keywords:** Mitochondria, Mitochondrial homeostasis, Mitochondrial diseases, Cancer, Therapy

## Abstract

Mitochondria are generally considered essential for life in eukaryotic organisms because they produce most of the energy or adenosine triphosphate (ATP) needed by the cell. Beyond energy production, it is now widely accepted that mitochondria also play a pivotal role in maintaining cellular homeostasis and signaling. The two core processes of mitochondrial dynamics, fission and fusion, serve as crucial foundations for maintaining mitochondrial morphology, distribution, and quantity, thereby ensuring cellular homeostasis. Mitochondrial autophagy (mitophagy) ensures the selective degradation of damaged mitochondria, maintaining quality control. Mitochondrial transport and communication further enhance their role in cellular processes. In addition, mitochondria are susceptible to damage, resulting in dysfunction and disruption of intracellular homeostasis, which is closely associated with the development of numerous diseases. These include mitochondrial diseases, neurodegenerative diseases, cardiovascular diseases (CVDs) and stroke, metabolic disorders such as diabetes mellitus, cancer, infectious diseases, and the aging process. Given the central role of mitochondria in disease pathology, there is a growing need to understand their mechanisms and develop targeted therapies. This review aims to provide a comprehensive overview of mitochondrial structure and functions, with a particular focus on their roles in disease development and the current therapeutic strategies targeting mitochondria. These strategies include mitochondrial-targeted antioxidants, modulation of mitochondrial dynamics and quality control, mitochondrial genome editing and genetic therapy, and mitochondrial transplantation. We also discuss the challenges currently facing mitochondrial research and highlight potential future directions for development. By summarizing the latest advancements and addressing gaps in knowledge, this review seeks to guide future research and clinical efforts in the field of mitochondrial medicine.

## Introduction

In the second half of the twentieth century, mitochondria were increasingly recognized as cellular energy factories. They consist of two separate lipid bilayer membranes and are a double-membrane organelle containing a wide variety of enzymes, particularly those involved in oxidative phosphorylation (OXPHOS) [1]. These enzymes catalyze the oxidative breakdown of organic matter, releasing energy and synthesizing adenosine triphosphate (ATP). This process provides the necessary energy for various cellular activities [2]. For decades, mitochondria's central role in energy metabolism was undisputed. However, recent research has revealed their multifunctionality, including involvement in signal transduction and cellular homeostasis [3, 4]. This expanded understanding highlights their complexity and the significant consequences of mitochondrial dysfunction.

When mitochondrial function is impaired, such as reduced ATP production, mitochondrial deoxyribonucleic acid (mtDNA) mutation, OXPHOS dysfunction, overproduction of mitochondrial reactive oxygen species (mtROS) and abnormal accumulation of some intermediates. It will lead to a cellular energy crisis and abnormal apoptosis, thus triggering a series of diseases [5–8]. These diseases include mitochondrial disorders, neurodegenerative diseases like Alzheimer's and Parkinson's, cardiovascular diseases (CVDs) and stroke, metabolic disorders such as diabetes mellitus, cancer, infectious diseases like coronavirus disease 2019 (COVID-19), and age-related decline. For example, primary mitochondrial disorders are genetic conditions that impair mitochondrial energy production [9]. Neurodegenerative diseases such as Alzheimer's and Parkinson's are also linked to mitochondrial impairments [10]. Damage to mtDNA can lead to uncontrollable inflammatory responses and cholesterol accumulation, subsequently resulting in the development of CVDs and stroke [11]. Metabolic disorders like diabetes mellitus, where insulin secretion from β-cells and insulin resistance (IR) are influenced by mitochondria [12]. Additionally, cancer cells often alter mitochondrial metabolism to support rapid proliferation [13]. Viral infections, such as COVID-19, can impair mitochondrial function and worsen disease severity [14]. Finally, the aging process itself is characterized by a decline in mitochondrial function, which is thought to contribute to the age-related increase in disease susceptibility and frailty [5, 15]. Given mitochondria's central role in these diseases, there has been a surge in the development of mitochondrial-targeted therapies. Mitochondria-targeted antioxidants can reduce oxidative stress caused by excessive reactive oxygen species (ROS) production [16]. Modulating mitochondrial dynamics can remove damaged mitochondria and promote healthy function. Quality control mechanisms are also being targeted to selectively degrade dysfunctional mitochondria and maintain a healthy mitochondrial population [6, 17, 18]. In more advanced therapeutic approaches, mitochondrial genome editing and genetic therapy are being explored to correct genetic mutations that cause mitochondrial diseases. This cutting-edge field holds the promise of treating the root cause of these disorders. Additionally, mitochondrial transplantation, the process of introducing healthy mitochondria into cells, is being investigated as a potential therapy for conditions where mitochondrial function is severely compromised [19–21].

In summary, this review provides a comprehensive overview of mitochondria, from their basic structure and functions to their roles in various diseases and the emerging therapeutic strategies targeting them. By highlighting the interplay between mitochondrial function and disease, and by discussing the latest therapeutic modalities, this review aims to contribute to the advancement of mitochondrial medicine and the betterment of patient outcomes.

## Basis of mitochondria

### Overview of mitochondrial structure and functions

Mitochondria originated from endosymbiotic alpha-proteobacterial and evolved from bacterial progenitor cells through symbiosis within eukaryotic host cells [22–24]. They are characterized by a double-membrane structure, consisting of an outer membrane and an inner membrane that encloses the intermembrane space (IMS) and the mitochondrial matrix. The inner membrane forms the cristae and embeds numerous enzymes and protein complexes. Beneath the inner membrane lies the mitochondrial matrix, which contains a circular genome known as mtDNA and several copies of ribosomes [25]. Unlike nuclear DNA (nDNA), mtDNA typically lacks intronic sequences and replicates independently of the host genome. Human mtDNA is approximately 16,569 base pairs long and encodes 37 genes. The light strand contains nine genes, eight of which encode tRNAs (Ala, Asn, Cys, Glu, Gln, Pro, Ser, Tyr), while the remaining gene encodes NADH-ubiquinone oxidoreductase chain (ND) 6. The heavy strand contains 28 genes. The classical transcriptional expression products include 13 proteins, 22 tRNAs and two rRNAs (12S and 16S) [26–28]. The 13 protein-coding genes are organized into two main clusters: one encoding subunits of respiratory complex I (ND1-6), another encoding subunits of respiratory complexes III cytochrome (cyt) B, IV (cyclo-oxygenase (COX) 1, COX2, and COX3), and V (ATP6 and ATP8). These proteins are essential components of the electron transport chain during OXPHOS [29]. MtDNA also contains several non-coding regions, such as the D-loop, which serves as the origin of replication and contains heavy strand promoter (HSP)1 and HSP2 and one light strand promoter (LSP) [29, 30]. This unique structure provides the physical and chemical environment necessary for mitochondria to perform their diverse functions.

### The role of mitochondrial in cellular homeostasis and signaling

Recent discoveries have shown that the mitochondria play many central roles in cells, beyond their primary energetic function. Mitochondria are now recognized as pivotal organelles for energy conversion, signal transduction, and regulation of cell death [3, 4, 31]. The core function of mitochondria is ATP generation through OXPHOS. In this process, cells extract electrons from nicotinamide adenine dinucleotide (NADH) and flavin adenine dinucleotide (FADH2) and use them to pump protons across the inner mitochondrial membrane (IMM), creating a proton gradient. This gradient drives ATP synthesis via ATP synthase, an enzyme embedded in the IMM. The electrons from NADH and FADH2 are passed through a series of protein complexes in the electron transport chain (ETC), ultimately combining with oxygen to form water, a process that is coupled with the proton gradient generation [32, 33]. The mitochondrial respiratory chain (MRC) is a key energy converter in eukaryotic cells, consisting of four complexes (I-IV) and two electron carriers embedded in the IMM [34]. The four complexes transfer electrons from various metabolic pathways to molecular oxygen, establishing an electrochemical gradient on both sides of the IMM to power ATP synthesis [2]. Complex I catalyzes the oxidation of reduced NADH and the reduction of ubiquinone with the contribution of two electrons and four protons, promoting the formation of a proton gradient across the IMM [35]. Complex II oxidizes succinic acid to fumarate and reduces ubiquinone to ubiquinol for electron transfer [36]. Complex III combines the oxidation of ubiquinol and the reduction of cyt C. It absorbs two protons and releases four protons during electron transfer [37]. Complex IV transfers the electrons cyt C releases to oxygen and reduces it to water. Two protons are transferred in this process against the concentration gradient. Additionally, Complex V (ATP synthase) comprises two functional domains, F0 and F1, using the proton gradient to catalyze ATP synthesis [38].

Mitochondrial signal transduction mechanisms are complex and diverse, covering a wide range of signal recognition and processing from the cell to the external environment, such as oxidative stress, mtROS signaling pathway, Ca^2+^ signaling, mitochondrial genome signaling, thermal signal transduction and inflammatory signaling [3, 39, 40]. Voltage-dependent anion channels (VDAC) in the outer mitochondrial membrane (OMM) mediate signal transduction between the cytoplasm and mitochondria [41]. Additionally, mitochondrial dynamics, including movement, fission, and fusion, are closely linked to these signaling pathways [42]. Mitochondria work in concert with the nucleus and multiple organelles to build a mitochondrial information processing system (MIPS), which can be summarized into three links. First, mitochondria have the ability to sense signals. These signals include changes in metabolite concentration, hormone signals, and oxidative stress. Secondly, integration of information and process signals through physical interaction and diffusion mechanisms. Finally, mitochondria produce output signals that regulate the function of other organelles and systemic physiology. These output signals can affect cell metabolism, gene expression, cell cycle, and other aspects, thus regulating of cell physiological activities [3].

## Mitochondrial dynamics and function

**Fig. 1:**
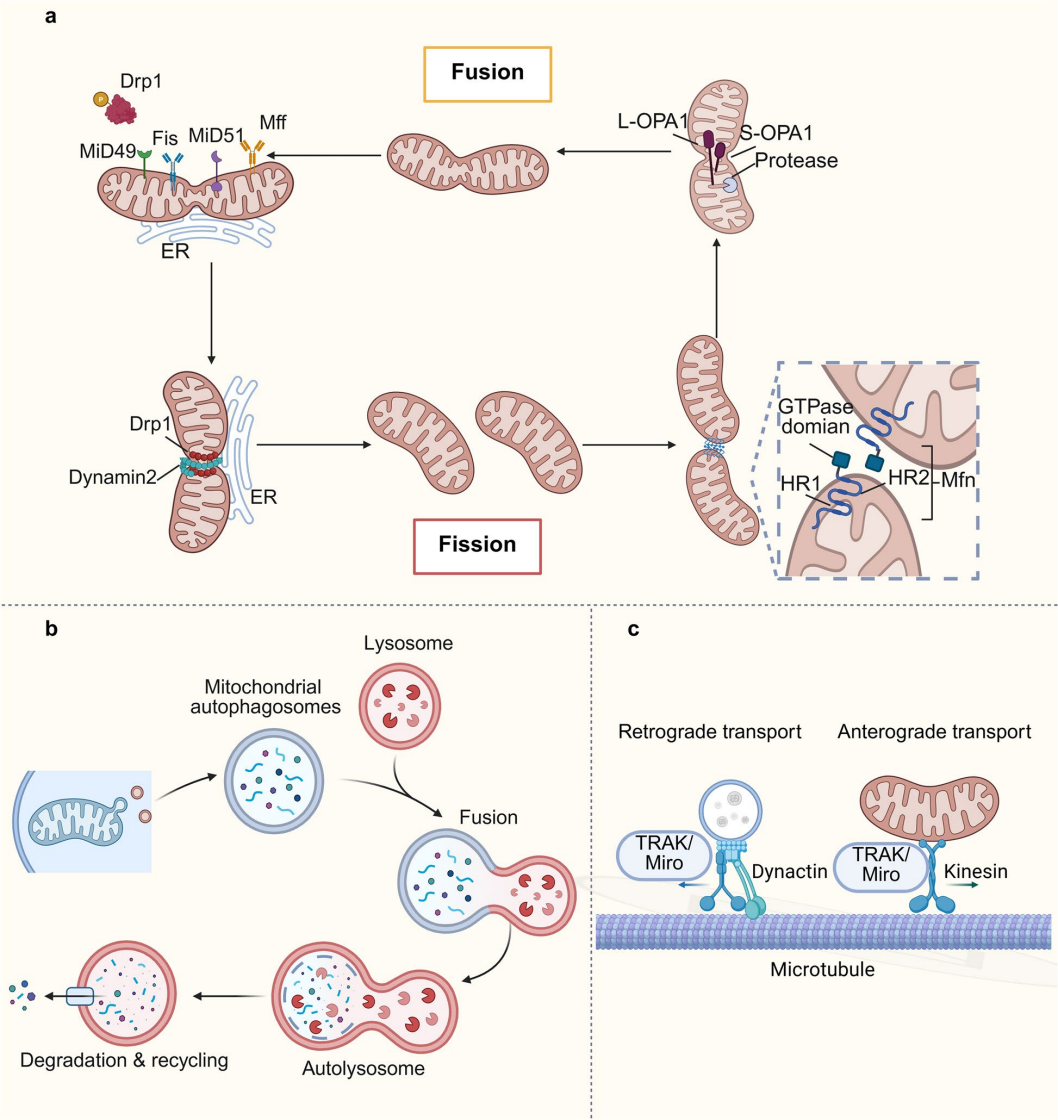
The mechanisms of mitochondrial dynamics. **a**. Mitochondrial fission and fusion. Initially, ER tubules come into contact with mitochondria to mediate constriction before Drp1 recruitment. Upon accumulation on the OMM, Drp1 forms a ring-like structure and undergoes GTP hydrolysis. Subsequently, dynamin2 gathers at the mitochondrial constriction site, where it assembles and completes the fission process, resulting in the formation of two daughter mitochondria. The binding and hydrolysis of GTP induce conformational changes in the GTPase domain of Mfn, leading to its oligomerization. This oligomerization of the GTPase domain causes the tethering of two mitochondria, facilitating their docking and fusion. Additionally, the oxidation of two cysteine residues located in the heptad-repeat 2 (HR2) domain of the Mfn molecule by increased levels of oxidized glutathione. OPA1, present in both S-OPA1 and L-OPA1 forms due to protease processing, is involved in the fusion of the IMM. **b**. Mitochondrial autophagosomes are formed and fuse with lysosome. The fusion of these organelles leads to the formation of autolysosome. The final step involves the degradation and recycling of mitochondria within the autolysosome. **c**. Mitochondria are transported along microtubules in both retrograde and anterograde directions. TRAK/Miro and dynactin are involved in retrograde transport, while TRAK/Miro and kinesin are involved in anterograde transport

Mitochondrial dynamics refers to the continuous process of fission and fusion within the cell, which regulates the distribution, quantity, size, and shape of mitochondria in a dynamic equilibrium. They encompassing the processes of fusion, fission, mitophagy, and mitochondrial transport, is a critical regulatory mechanism that maintains the functional integrity and metabolic homeostasis of mitochondria within cells [43].The mechanisms of mitochondrial dynamics have been summarized in Fig. 1.

### Fission and fusion

Fission, the process by which mitochondria divide into smaller units, is mediated by the GTPase dynamin-related protein 1 (Drp1). Drp1 interacts with four OMM-binding proteins, including mitochondrial fission 1 (Fis1), mitochondrial fission factor (Mff), mitochondrial dynamics protein (MiD) 49, and MiD51 [43]. Drp1 and Fis1 are the most critical proteins involved in mitochondrial division. Conversely, mitochondrial fusion is driven by mitofusin 1 (Mfn1) and mitofusin 2 (Mfn2), which mediate outer membrane fusion, while inner membrane fusion is regulated by optic atrophy protein 1 (OPA1) [44]. Drp1 is recruited to mitochondrial constriction sites, often facilitated by interactions with actin and the endoplasmic reticulum (ER), leading to the formation of two daughter mitochondria. Mitochondria-specific lipid cardiolipin activates Drp1 oligomerization, promoting the formation of helical structures and enhancing GTPase activity. The nucleotide-driven conformational changes in Drp1 not only facilitate its assembly but also drive disassembly processes, collectively inducing mitochondrial fission [43].

Conversely, mitochondrial fusion involves the merging of mitochondria to form a network and is driven by two distinct events: outer membrane fusion, mediated by Mfn1 and Mfn2, and inner membrane fusion, regulated by OPA1 [44]. The presence of both Mfn1 and Mfn2 significantly enhances fusion efficiency. Specifically, Mfn1 deficiency leads to marked mitochondrial fragmentation, while Mfn2 deficiency results in a higher proportion of mitochondria with spherical or ovoid shapes [45]. Moreover, mitochondrial dynamics integrate into various physiological activities and signal transduction cascades by regulating the post-translational modifications of Mfn proteins [46]. Inner membrane fusion is primarily regulated by OPA1, a dynamin-like GTPase, which exists in two splice variants: long OPA1 (L-OPA1) and short OPA1 (S-OPA1). The balance between these two forms determines mitochondrial fusion capacity [47]. In addition, OPA1 also coordinates mitochondrial cristae integrity and oxidative OXPHOS processes, thereby preventing apoptosis [48]. The balance between fission and fusion determines mitochondrial morphology and adapts it to cellular metabolic needs (Fig. 1a) [49].

### Mitophagy and mitochondrial quality control

Mitochondrial autophagy (mitophagy) is a selective autophagy process that targets damaged or dysfunctional mitochondria for degradation, playing a pivotal role in mitochondrial quality control (MQC) (Fig. 1b) [50]. When mitochondria are damaged or ischaemia or hypoxia, mtDNA mutations gradually accumulate under the effect of ROS, resulting in reduced intracellular mitochondrial membrane potential (ΔΨm) and depolarized damage, which triggers mitochondrial autophagy [51]. The molecular mechanisms of mitophagy include the ubiquitin (Ub)-dependent pathways and the Ub-independent pathways. Among them, the phosphatase and tensin homolog (PTEN) induced putative kinase 1 (PINK1)/Parkin pathway is one of the most widely studied Ub-dependent pathways [52]. In damaged mitochondria, the depolarization of the IMM prevents the degradation of PINK1, leading to the stable accumulation of full-length PINK1 in the OMM. Subsequently, PINK1 recruits Parkin to the OMM and stimulates its E3 ubiquitin ligase activity by phosphorylating ubiquitin at Ser65. Parkin then ubiquitinates multiple OMM proteins such as Mfn1, Mfn2, and VDAC1, and these labeled proteins become signals for autophagosome recognition and encapsulation, facilitating the initiation of mitophagy [52, 53]. In addition, the Ub-independent pathway involves some mitophagy receptors localised to the OMM, such as FUN14 domain containing 1 (FUNDC1), B cell lymphoma-2 (BCL-2), adenovirus E1B 19 kDa interacting protein 3 (BNIP3), Nip3-like protein X (NIX), BCL-2-like protein 13 (BCL2L13) and FK506 binding protein 8 (FKBP8) [54–56]. These receptors induce mitochondrial clearance by binding to the microtubule-associated protein light chain 3 (LC3) via the LC3 interaction region (LIR) [57]. Their phosphorylation state and ability to bind to LC3 are affected by a variety of kinases like casein kinase 2 (CK2), SRC, Unc-51-like autophagy activating kinase 1 (ULK1), c-Jun N-terminal kinase (JNK) 1/2, protein kinase cAMP-activated (PRKA), phosphatases such as phosphoglycerate mutase 5 (PGAM5) and multiple stimuli [58–60]. Also, the E3 ligase membrane-associated RING Finger protein 5 (MARCH5) and the deubiquitinating enzyme ubiquitin-specific peptidase 19 (USP19) perform critical roles in regulating FUNDC1 protein levels and mitochondrial fission activity [61]. Hypoxia-inducible factor (HIF)−1α controls the expression of receptors BNIP3 and NIX, which is a crucial factor in inducing mitophagy under hypoxic conditions [62]. Notably, the autophagy essential proteins autophagy-related 9 A (ATG9 A) and 200kD focal adhesion kinase (FAK) family kinase-interacting protein (FIP200) are dependent on autophagy junction proteins such as optineurin (OPTN) and nuclear dot protein 52 (NDP52) for recruitment on damaged mitochondria [63–65]. These proteins are recruited to the OMM by recognising phosphorylated poly-Ub chains. Phosphorylation of OPTN by TANK-binding kinase 1 (TBK1) enhances its capacity to bind to poly-Ub chains, thus creating a positive feedback cycle that promotes mitophagy [66, 67].

**Fig. 2:**
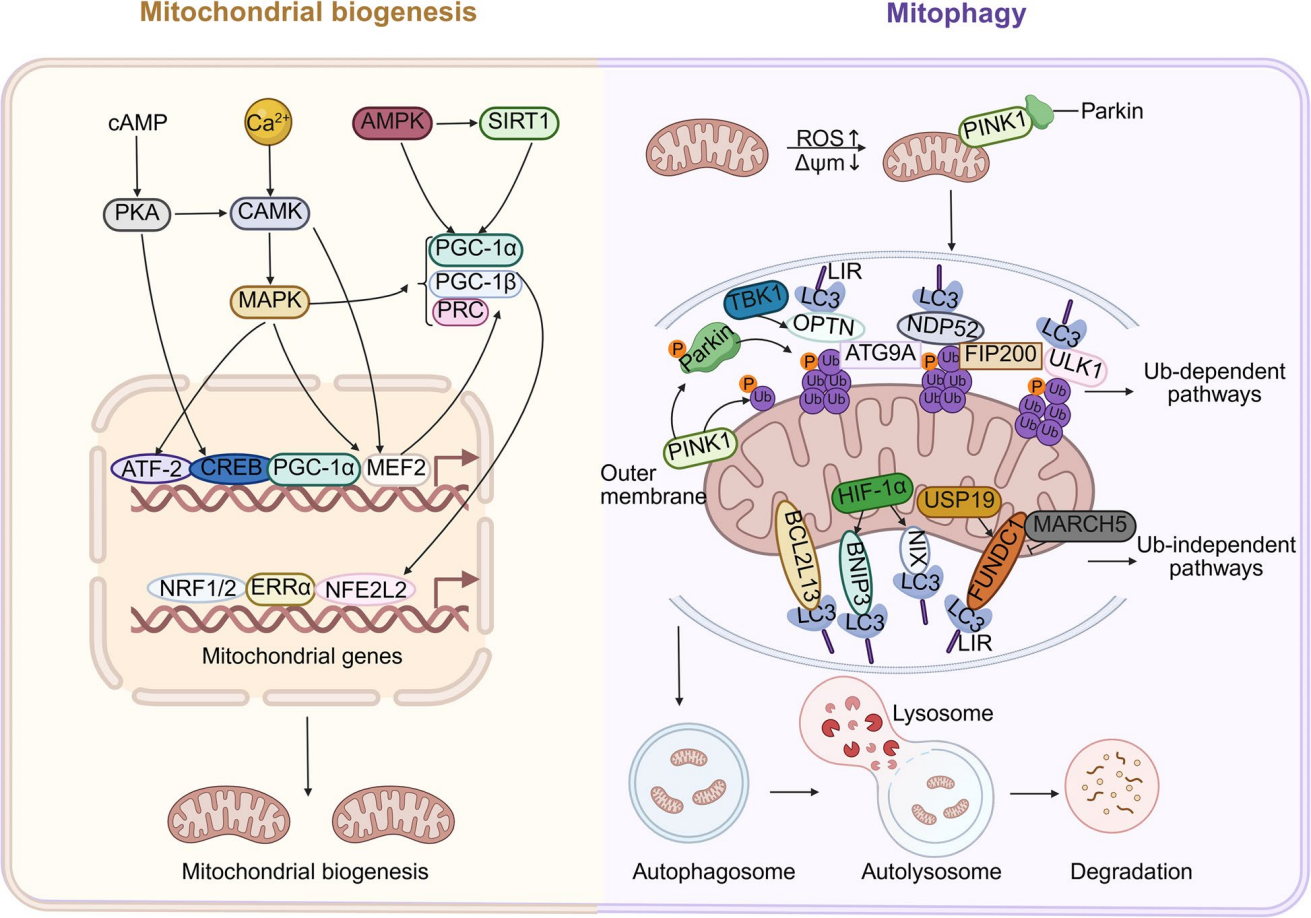
Mitochondrial homeostasis is regulated by mitochondrial biogenesis and mitochondrial autophagy. cAMP activates PKA, which then influences the activity of transcription factors that control mitochondrial gene expression. Ca^2+^ trigger the activation of CAMK, which in turn activates MAPK. This pathway also contributes to the regulation of mitochondrial gene expression. AMPK activates SIRT1, which influences the expression of PGC-1α, PGC-1β, and PRC. These factors are crucial for the regulation of mitochondrial biogenesis. The transcription factors ATF-2, CREB, PGC-1α, and MEF2 are activated by the signaling pathways and work together to regulate the expression of mitochondrial genes. Additionally, NRF1/2, ERRα, and NFE2L2 are involved in the regulation of mitochondrial gene expression. The activation of these transcription factors leads to the expression of mitochondrial genes, which are essential for the biogenesis of new mitochondria. Mitophagy involves both ubiquitin-dependent and ubiquitin-independent pathways. In the ubiquitin-dependent pathway, PINK1 stabilizes on the outer mitochondrial membrane, leading to the recruitment of Parkin, which ubiquitinates mitochondrial proteins. Key proteins include TBK1, OPTN, NDP52, FIP200, ULK1, ATG9 A, and LC3. In the ubiquitin-independent pathway, proteins like BNIP3 and NIX are involved. Autophagosomes form around mitochondria, fuse with lysosomes to form autolysosomes, leading to mitochondrial degradation

Mitochondrial biogenesis is another critical component of MQC, involving DNA replication, transcription, translation, and phospholipid transport between organelles. MtDNA replication and transcription occur in the mitochondrial matrix, while mitochondrial proteins encoded by nuclear DNA (nDNA) are synthesized in the cytoplasm and imported into mitochondria via a molecular chaperone system [68, 69].To be specific, mtDNA replication and transcription occur mainly in the mitochondrial matrix, while mitochondrial proteins encoded by nuclear DNA (nDNA) are synthesized in the cytoplasm and imported into mitochondria via a molecular chaperone system [68, 69]. Mitochondrial retrograde signals such as ATP levels, ROS and Ca^2+^ modulate intracellular signaling cascades, affecting mitochondrial biogenesis [70]. The peroxisome proliferator-activated receptor γ (PPARγ) coactivator-1alpha (PGC-1α) has been regarded as a major regulator of mitochondrial biogenesis. The coactivators PGC-1α, PGC-1β and PPARγ-related coactivator 1 (PRC) bind to the critical transcription factors nuclear respiratory factors 1 (NRF1) and 2 (NRF2), estrogen-related receptor α (ERRα) and nuclear factor erythroid 2-like 2 (NFE2L2) to accelerate the transcription of biogenesis-related genes [69, 71]. Moreover, transcription factors like the cyclic adenosine monophosphate (cAMP) response element-binding protein (CREB), the myocyte enhancer factor 2 (MEF2) and activating transcription factor-2 (ATF-2) work together to promote PGC-1α expression [17]. Mitochondrial biogenesis is controlled by various signaling pathways. Specifically, the AMP-activated protein kinase (AMPK)/sirtuin 1 (SIRT1)/PGC-1α system regulates PGC-1α activity and expression according to changes in intracellular energy levels [72]. The Ca^2+^/calmodulin-dependent protein kinase (CAMK)/p38 mitogen-activated protein kinase (MAPK)/PGC-1α pathway senses changes in intracellular calcium ion concentration and the cAMP-protein kinase A (PKA) pathway senses changes in cAMP levels to regulate mitochondrial biogenesis [17, 73]. Through the biogenesis process, cells continuously produce new, healthy mitochondria to replace old or damaged ones (Fig. 2).

### Mitochondrial transport

In highly polarized cells like neurons, mitochondrial transport is essential [74]. Mitochondria move along microtubules, facilitated by the trafficking kinesin-binding protein (TRAK)/mitochondrial Rho GTPase (Miro) motor adapter complex. This complex ensures that mitochondria are positioned at sites of high energy demand and enables communication with other cellular components [75, 76]. Furthermore, mitochondria engage in dynamic interactions with the cellular milieu, including the formation of tunneling nanotubes (TNTs) that facilitate direct intercellular transfer of organelles like mitochondria [77]. This transfer is crucial for mitochondrial quality control, enabling recipient cells to replenish damaged mitochondria and degrade dysfunctional ones (Fig. 1c). Additionally, mitochondria communicate with other organelles, such as the ER and peroxisomes, to regulate calcium levels and metabolic intermediates essential for cellular signaling and energy metabolism [78, 79].

In summary, mitochondrial dynamics is a highly regulated process central to mitochondrial and cellular function. Understanding the molecular mechanisms governing these dynamics is crucial for elucidating how mitochondrial shape meets function and for advancing our knowledge of diseases associated with mitochondrial morphology defects.

## The role of mitochondria in disease

This section examines the pivotal role of mitochondria in a range of diseases, emphasizing their impact on disease progression. Highlighted are neurodegenerative diseases, metabolic disorders and cancer, where mitochondrial function plays a critical part. We will discuss how mitochondrial dynamics and autophagy influence these conditions and explore therapeutic strategies that target mitochondrial health. Additionally, we'll consider the broader implications of mitochondrial decline in the context of aging.

### Mitochondrial diseases

Mitochondrial diseases are a group of most common heterogeneous inherited metabolic disorders, featured by genetic deficiencies in OXPHOS and ATP synthesis, the clinical expression of which shows significant variability [9]. As mentioned above, mitochondrial function is uniquely under the dual control of two different genomes, mtDNA and nDNA [80]. This dual genetic control accounts for the diverse inheritance patterns observed, ranging from maternal inheritance of mtDNA mutations to autosomal recessive, dominant, or X-linked inheritance of nDNA mutations [26, 81]. Pathogenic mtDNA mutations primarily fall into three categories: point mutations in protein-coding genes (mRNA), mutations in genes involved in protein synthesis (tRNA or rRNA), and mtDNA rearrangements. These mutations lead to various clinical symptoms depending on the proportion of mutant and wild-type mtDNA (heteroplasmy) within each cell [82]. This implies that a critical threshold of mtDNA mutations must be exceeded before biochemical defects in the respiratory chain become detectable [83]. Besides, the clinical manifestations are very wide-ranging, presenting both oligo-symptomatic states and complex multisystem syndromes containing a range of neurological and non-neurological symptoms [81].

Leber hereditary optic neuropathy (LHON) is the typical single organ involvement mitochondrial disorder caused by mtDNA point mutations such as genevariation on m.3460G → A MT-ND1, m.11778G → A MT-ND4, and m.14484 T → C MT-ND6. The genetic mechanism is the maternal inheritance. These mutations affect Complex I of the mitochondrial respiratory chain, leading to impaired energy metabolism. LHON primarily manifests as acute or subacute optic neuropathy, causing vision loss, especially in young men [84]. To date, redox modulator idebenone is widely used in treating of patients with LHON and appears to have positive results with prolonged use [85]. In addition, some pre-clinical/clinical gene therapy studies based on allotopic expression offer promising approaches for LHON [86, 87]. Another retinopathy disease, Kearns-Sayre syndrome (KSS), is caused by the deletion of large segments of mtDNA [88]. These deletions involve multiple genes encoding the mitochondrial respiratory chain components, resulting in an inadequate energy supply. The mechanism of inheritance is also maternal. The main symptoms of KSS include progressive extraocular muscle paralysis, vision loss due to retinitis pigmentosa, heart block, cerebellar ataxia, and cognitive deficits [87].

Mitochondrial encephalomyopathy lactic acidosis and strokelike episodes (MELAS) syndrome is primarily caused by specific point mutations in mtDNA. The most common mutation is m.3243 A → G in the mitochondrial tRNA leucine 1 (MT-TL1) gene which encodes mitochondrial tRNA [89, 90]. These mutations impair mitochondrial protein synthesis, affecting energy metabolism, especially in tissues with high energy requirements, such as brain and muscle. MELAS syndrome is inherited maternally. Patients with MELAS syndrome usually present with recurrent stroke-like episodes, seizures, muscle weakness, lactic acidosis, and mental retardation [89]. L-Arg supplementation has been used to treat acute episodes. However, the rarity of the disease makes it challenging to conduct sufficient clinical studies. More data are needed to confirm the exact mechanism by which L-Arg supplementation affects patients with MELAS syndrome [91].

To conclude, mutations in any one of these mitochondrial or nuclear genes disrupt mitochondrial function, leading to defects in OXPHOS. These defects manifest in various organs with diverse symptoms. Current therapies mainly focus on alleviating symptoms rather than addressing the root cause, such as a specific mutation. Encouragingly, the establishment of large patient cohorts and the recent application of advanced techniques particularly whole-genome sequencing (WGS), have broadened the landscape of known disease-associated mutation genes, holds promise for a diagnostic revolution in mitochondrial diseases [92, 93]. Mitochondrial dysfunction in various diseases is summarized in Table 1.

**Table 1:** Diseases related with mitochondria

Diseases	Mitochondrial dysfunction	Outcomes	Reference
Mitochondrial diseases			
Leber hereditary optic neuropathy	MtDNA point mutations	Affect the function of the mitochondrial respiratory chain complex, particularly Complex I, resulting in disturbed energy metabolism	[84]
Kearns-Sayre syndrome	Deletion of large segments of mtDNA	Deletions involve multiple genes encoding components of the mitochondrial respiratory chain, resulting in inadequate energy supply	[88]
Mitochondrial encephalomyopathy lactic acidosis and strokelike episodes	MtDNA point mutations	Mutations cause impaired mitochondrial protein synthesis, which in turn affects energy metabolism	[89, 163]
Neurodegenerative Diseases			
Alzheimer’s disease	Impaired energy metabolism	Interact with the aggregated protein including Aβ and tau in AD leads to the loss of synaptic function associated with learning and memory	[104, 109]
	Impaired calcium homeostasis	Cause the pathology associated with AD	[105, 106]
	Increased levels of Drp1 and Fis1 and reduced expression levels of OPA1, Mfn1, and Mfn2	Impaired balance of fission and fusion promotes mitochondrial fragmentation and causes overproduced ROS	[110, 111]
Parkinson’s disease	Respiratory chain complex dysfunction	Complexes I, IV and the subunits of all five OXPHOS complexes decreased in PD	[123]
	Loss of function mutations in Parkin and PINK1	Accounting for autosomal recessive forms of early-onset PD and associated with degradation of damaged mitochondria	[128]
	Interaction of mitochondrial damage with α-syn accumulation	Accumulated α-syn in mitochondrial causes reduced mitochondrial Complex I activity, accompanied with increased oxidative stress, and inflammation in dopamine neurons	[131, 132]
Atherosclerosis	Caspase-1-mediated mitochondrial damage	MtROS production, mitochondrial swelling, fragmentation etc., particularly also inhibiting mitophagy to amplify mitochondrial damage	[11]
	Internalize of oxLDL and formation of foam cells	Pathological changes in macrophages, including inflammatory state and polarization	[143, 157]
	Increased IL-6 levels	Enhance mitophagy and elevate Parkin levels	[144]
	Decreasing synthesis and secretion of NO	Feedback amplification system forms a vicious cycle, contributing to ECs dysfunction and atherogenesis	[153]
Stroke	Calcium overload, opening of mPTP, and ATP depletion	Disrupts intracellular signaling, initiating a cascade involving ER stress, oxidative stress, inflammation, and autophagy	[143, 157, 164]
	High cerebrospinal fluid lactate levels	Neuronal death after stroke	[165]
Metabolic disorders			
Diabetes mellitus	Down-regulation of Drp1	Morphological changes, accompanied with mitochondrial membrane potential and ATP generation, leading to significantly impaired GSIS	[172]
	Abnormalities of mitochondrial number, density, morphology and function	Mitochondria-originated ROS impairs insulin-stimulated glucose uptake and GLUT4 translocation, significantly resulting IR	[179]
	Disruption of ER-mitochondria interactions and calcium exchange	Impair hepatic insulin action and lipid-related mitochondrial oxidative metabolism	[181]
Liver diseases			
Alcoholic liver disease	Inhibition the synthesis of mitochondrial respiratory complex proteins	Reduce OXPHOS capacity, increase ROS levels, and trigger oxidative stress	[190]
	Depletion of the Parkin gene	Impaired mitophagy accelerates alcohol-induced liver injury and fatty degeneration	[192]
Non-alcoholic fatty liver disease	Hepatocytes have increased expression of proteins involved in mitochondrial fusion and fatty degeneration of the liver	Systemic metabolic disorders	[193]
	Mitochondrial enlargement and reduced activity of the respiratory chain enzyme complex	Hepatic lipid metabolism and hepatic damage	[194]
Metabolic dysfunction-associated steatotic liver disease	Increased mtROS generation	Hepatic fat accumulation, defective insulin signaling and dysregulated lipoprotein transport	[196, 197]
Hepatic ischaemia–reperfusion injury	Impaired autophagy capacity	Ineffective clearance of damaged mitochondria	[198]
Cancer	MtDNA mutations	Alter mitochondrial metabolism, enhance tumorigenesis, and enable cancer cells to adapt to the changing environment	[7, 207]
	Mutations in nDNA genes	Inhibit α-ketoglutarate-dependent dioxygenases and activate the NRF2 stress pathway which promotes tumorigenesis	[211]
	Altered mitochondrial metabolism	Increase mtROS production and change cellular redox status. It affects the activity of factors such as HIF-1α and AP-1, thereby altering gene expression and stimulating cancer cells proliferation	[212, 213]
	Decrease in mitochondrial membrane potential	Reduce mitochondrial Ca^2+^ uptake and limit the activation of the mitochondrial apoptotic pathway	[214]
Infectious diseases			
COVID-19	Metabolically impaired, with decreased respiration and glycolysis	Decreased basal and maximal respiration, lower spare respiratory capacity and that leads to redistribution of monocyte subsets, associated with the exaggerated inflammation in pneumonia	[237]
	Reduced energy production, increased oxidative stress, and mitochondrial damage	Produce significantly elevated ROS, trigger the stimulation of additional pro-inflammatory cytokine, eventually result a cytokine storm	[235, 238, 241]
	Release of mtDNA	The onset of multi-organ failure in acute respiratory distress patients	[242]
Viral hepatitis	Infected by HCV	Alter mitochondrial dynamics, promotes mitophagy, diminishes the IFN response and contributes to viral replication and persistent infection	[244]
	Infected by HBV	Dlevated levels of ROS exacerbating the exhaustion of virus-specific CD8 + T cells, manifested as increased protein oxidation and DNA damage	[245]
Aging	Decreased mitochondrial ADP sensitivity	Increase mitochondrial H_2_O_2_ emissions causing age-related redox stress	[251]
	MtDNA point mutations and deletions accumulate	Influence energy consumption and lead to oxidative damage	[250]
	Decreased efficiency of OXPHOS	Increased ROS causes oxidative damage to mitochondrial DNA, proteins, and lipids, further reducing mitochondrial dynamics and hindering mitophagy	[15, 252, 253]

### Neurodegenerative disorders

Neurodegenerative diseases are a heterogeneous group of age-related disorders featured by progressive loss of selective vulnerable neuronal populations and accumulation of altered proteins. Typical examples include Parkinson's (PD), Alzheimer's (AD), amyotrophic lateral sclerosis (ALS) and Huntington's (HD) disease [94]. Mitochondria are essential regulators of neurogenesis, and the main functions of mitochondria, including ATP production, Ca^2+^ buffering capacity, ROS production and the regulation of apoptosis, have been suggested to be involved in the pathogenesis of neurodegeneration [5, 95]. Additionally, specific interactions between disease-related proteins and mitochondria contribute to the progression of these disorders [96]. Undoubtedly, understanding the link between mitochondrial dysfunction and neurodegeneration is crucial for developing new therapies. Here, we summarize the role of mitochondria in several typical progressive neurodegenerative diseases.

#### Alzheimer’s disease

Alzheimer's disease (AD), the most prevalent neurodegenerative disease, is characterized by two pathological hallmarks: the aggregation of insoluble forms of amyloid β (Aβ) in extracellular plaques, as well as the deposition of neurofibrillary tangles (NFTs) made up mainly of hyperphosphorylated microtubule protein tau in neurons [97–99]. Abundant evidence demonstrates that mitochondria dysfunction plays a fundamental role in the pathogenesis of AD. Mitochondrial abnormalities and oxidative stress as an early and prominent feature of AD [100].

Impaired energy metabolism is a hallmark of the AD brain, consistent with disrupted mitochondrial bioenergetics. This includes downregulation of mitochondrial OXPHOS, decreased expression of mitochondrial complexes I-V, and dysfunction of respiratory enzymes in AD brains and models [101]. Synaptic hypometabolism has been linked to cognitive impairments and drives AD progression [102]. Moreover, accompanied by less efficient production of ATP, damaged mitochondria are more efficient producers of ROS [103], and this oxidative stress could account for the loss of synaptic functions associated with learning and memory in AD [104]. Impaired calcium homeostasis, another central event in AD, is also related to mitochondria. Targeting mitochondrial Na^+^/Ca^2+^ exchanger (NCLX) to modulate Ca^2+^ load in neurons provides a new strategy for impeding AD-associated pathology [105, 106].

Mitophagy, a key component of MQC, is impaired in the hippocampus of AD patients [107]. Furthermore, the mutations of either PINK1 or Parkin have been mentioned to be involved in early-onset familial PD [108]. Martín-Maestro, P et al. suggested that both Aβ and p-tau pathologies contribute to defective mitophagy, while lead to the Aβ and p-tau accumulation. Interestingly, restoration of neuronal mitophagy ameliorated Aβ pathology and abolished AD-related tau hyperphosphorylation, reversing memory impairment and cognitive deficits in vivo [109].

Abnormal mitochondrial dynamics, which control morphology, number, and distribution through fission and fusion, also play a role in AD. Increased levels of fission genes (e.g., Drp1 and Fis1) and decreased levels of fusion genes (e.g., OPA1, Mfn1, and Mfn2) promote mitochondrial fragmentation and ROS overproduction [110, 111]. Additionally, oligomeric Aβ and hyperphosphorylated tau could interact with Drp1, inducing excessive mitochondrial fragmentation and ultimately contributing to neuronal damage and cognitive deficits in AD [112, 113].

In a word, robust evidence highlights the essential role of mitochondria in AD onset and progression. Enhancing mitochondrial function may delay pathological processes and is crucial for developing new therapeutic interventions [10].

#### Parkinson’s disease

Parkinson's disease (PD) is the second most common neurodegenerative disorder, characterized by motor symptoms such as bradykinesia, rigidity, rest tremor, and postural instability. These symptoms are associated with the progressive loss of dopaminergic neurons in the substantia nigra (SN) pars compacta (SNpc) and the presence of lewy bodies, composed of α-syn aggregates [114, 115]. Many lines of evidence strongly suggest the involvement of mitochondria in neurodegenerative diseases [116, 117]. Noteworthily, mitochondria have been supported to play a particularly striking role in PD as an initiator or propagator [118, 119].

The link between mitochondria and PD was first reported in 1983 when 1-Methyl-4-phenyl-1,2,5,6-tetrahydropyridine (MPTP), a neurotoxin that interferes with respiratory chain complexes, was found to cause parkinsonian-like symptoms in drug users [120]. Since then, mitochondria initially have become the focus of the PD. Meanwhile, MPTP is now considered one of the most used neurotoxins in modelling PD in animals [121, 122]. Furthermore, a recently in-depth proteomic profile of the mitochondrial complexes within single neurons in PD patients, polymerase gamma (POLG) mutations and controls reveals that not only complexes I and IV but the subunits of all five OXPHOS complexes decreased in PD, highlighting the importance of individual respiratory capacity in neurodegeneration [123].

Multiple mtDNA deletions and copy-number variations are also implicated in PD. Deletions and copy-number regulation are the most common forms of defects in PD. These defects are common in aged individuals, although their role as causes or consequences of PD remains unclear [124]. Decades of research in the field confirmed that a striking number of genes associated with familial PD are directly or indirectly involved in the regulation of mitochondrial function, including PINK1, Parkin, DJ-1, leucine-rich repeat kinase 2 (LRRK2), α-syn, ubiquitin carboxyl-terminal hydrolase ligase-1 (UCHL-1), nuclear receptor-related 1 (NURR1), vacuolar protein sorting 13 C (VPS13 C), and high-temperature demand protein A2 (HTRA2) [125–127]. Among them, PINK1 and Parkin are the most extensively studied examples, which account for autosomal recessive forms of early-onset PD and are associated with the degradation of damaged mitochondria [128]. They play crucial roles in mitochondrial quality control, affecting fission–fusion balance, transport, and biogenesis, all of which are linked to PD development [129].

Growing evidence supports that the interplay between mitochondrial damage and accumulated α-syn is another way mitochondria mediate the pathology of PD [130]. It has been demonstrated that mitochondria could target α-syn due to the presence of a cryptic mitochondrial targeting signal in the N-terminal 32-amino acid region. Accumulated α-syn in mitochondria causes reduced mitochondrial Complex I activity, accompanied by increased oxidative stress and inflammation in dopamine neurons [131, 132]. Moreover, α-syn aggregates have been proposed to interfere with mitochondrial function in other mechanisms. Direct interaction with the mitochondrial membrane induces mitochondrial fragmentation, leading to decreased respiration and neuronal death [133]. Aggregation of α-syn and dysregulation of PGC-1α may form an interacting vicious circle, with increased α-syn oligomerization in PGC-1α reference gene (RG-PGC-1α)-deficient neuronal cells [134]. Accumulated α-syn in the ageing brain may impair mtDNA transcription and repair through signaling from the nucleus to the mitochondria [135].

In summary, extensive research has clarified the role of mitochondria in PD, indicating that targeting mitochondrial is a promising therapeutic strategy.

Similar to AD and PD, accumulating evidence suggests that compromised mitophagy lead to the progression of other neurodegenerative diseases, such as HD, ALS, and spinal muscular atrophy (SMA) [136–138]. Despite the heterogeneity of this group of diseases, mitochondria seem to be a pivotal crossroads in the biological processes promoting neurodegeneration as well as predicting clinical outcomes [10, 108]. Thus, investigating novel mitochondrial-targeted therapies holds promise for treating various neurodegenerative disease.

### Cardiovascular diseases and stroke

#### Cardiovascular diseases

CVDs, a leading cause of death and morbidity worldwide, encompass a range of conditions including heart failure, myocardial hypertrophy, atherosclerosis, cardiomyopathy, and others [43, 139]. Among these, atherosclerosis is the most extensively studied in mitochondrial research. It is a complex chronic inflammatory disorder characterized by the retention of plasma lipoproteins, particularly low-density lipoprotein (LDL), in the subendothelial space. This process involves vascular endothelial cells (ECs), vascular smooth muscle cells (VSMCs), and the immune system [140–142]. Emerging evidence highlights mitochondria as an important factor in the initiation and progression of atherosclerosis through multiple mechanisms, including energy metabolism, ROS production, release of mitochondrial components, and regulation of inflammation [143, 144].

The development of inflammatory response and the maintenance of chronic inflammation are key pathology characteristics in atherosclerosis [145]. Free mtDNA acts as an endogenous damage-associated molecular patterns (DAMPs) recognized by pattern-recognition receptors (PRRs), triggering inflammatory responses. This includes the activation of nuclear factor-κB (NF-κB), which promotes the expression of inflammatory mediators such as tumor necrosis factor-α (TNF-α) and interleukin (IL)−6. Additionally, mtDNA in vascular cells activates the NOD-like receptor protein 3 (NLRP3) inflammasome, leading to the activation of pro-inflammatory cytokines IL-18 and IL-1β [146, 147]. Meanwhile, increased mtROS generation also contributes directly or indirectly to the stimulation of NLRP3 inflammasomes [148]. Intriguingly, the interplay between mitochondria and the NLRP3 inflammasome is not unidirectional. NLRP3 inflammasome activation triggers Caspase-1-mediated mitochondrial damage, characterized by increased mtROS production, mitochondrial swelling, and fragmentation, while also inhibiting mitophagy and amplifying mitochondrial damage [11]. This creates a vicious cycle that exacerbates inflammation and mitochondrial dysfunction, ultimately contributing to atheroma formation. Moreover, age-related increased IL-6 levels prime the vasculature to exacerbate atherogenesis ahead of hyperlipidemia through enhancing mitophagy and elevating Parkin levels [144].

ECs line most blood vessels and play crucial roles in maintaining vascular homeostasis, regulating vascular wall permeability, and controlling cellular transport [149]. Generally, mitochondrial content in ECs takes merely 2–6% of cytoplasm volume and is relatively low in comparison to other cell types [150]. Correspondingly, instead of mainly functioning as an energy provider, the mitochondria of ECs are mostly involved in nitric oxide (NO) production, intracellular signaling and the senescence and apoptosis of endothelial cells, all of which are associated with atherosclerosis development. Reduced NO secretion is a hallmark of early EC dysfunction due to inactivated endothelial NO synthase (eNOS). Oxidized LDL (oxLDL), angiotensin-II, hyperglycemia, and hypoxia can increase mtROS, leading to eNOS degradation and reduced NO synthesis and secretion [151, 152]. In return, dysfunctional eNOS will produce more ROS, and this feedback amplification system forms a vicious cycle, contributing to ECs dysfunction and atherogenesis [153]. Peroxisome PGC-1α is the main regulator of mitochondrial biosynthesis and dynamics. Researches show that the overexpression of PGC-1α promoting mitochondrial biogenesis may prevent or treat atherosclerosis in multiple mechanisms, including regulating ROS generation and apoptosis of ECs, promoting NO production, decreasing the activity of NF-κB [154].

Macrophages are the predominant cells in all stages of the disease, involving inflammation response, cholesterol accumulation and plaque formation. Mitochondrial damage contributes to pathological changes in macrophages, including inflammatory state and polarization [155, 156]. Macrophages take up lipids and transform into foam cells, while mtDNA acts as DAMPs recognized by PRRs, promoting oxLDL internalization. Additionally, mitochondrial functioning plays an important role in the dynamics of macrophage polarization. M1 macrophages are the major subtype in unstable human plaques, which have strong phagocytosis and the ability to release pro-inflammatory cytokines. In contrast, M2 macrophages, found in stable plaques, reduce inflammation and promote tissue repair [157]. MtROS has exhibited the ability to induce the polarization of macrophage to M1 [143]. Accordingly, it could be an attractive research topic to regulate mitochondrial metabolism in macrophages, thus regulating mitochondrial metabolism in macrophages to inhibit M1 polarization could stabilize plaques and slow atherosclerosis progression [158]. Therapies directly targeting mitochondria such as mitochondrial antioxidants Mitoquinone (MitoQ) and mitochondrial dynamic modifier have a broad application prospect in the treatment of atherosclerosis [159, 160].

#### Stroke

Stroke is a leading cause of death and disability worldwide. The majority of strokes are ischemic, typically caused by blockages in the cerebral blood vessels. Inadequate blood supply deprives brain cells of necessary glucose and oxygen, disrupting cellular homeostasis and ultimately leading to neuronal damage and death [161, 162]. Mitochondria play a central role in this cascade of events and are implicated in the progression of brain injury induced by stroke. Additionally, mitochondrial disorders such as MELAS syndrome can cause non-vascular acute neurological dysfunction, leading to stroke-like episodes [163].

Neuronal death following stroke is exacerbated by calcium overload, the opening of the mitochondrial permeability transition pore (mPTP), and excessive production of ROS, all of which are consequences of impaired mitochondrial function. ATP depletion post-stroke disrupts intracellular signaling, initiating a cascade involving ER stress, oxidative stress, inflammation, and autophagy. Mfn2-mediated mitochondrial dynamics in megakaryocytes affects platelet function, influencing stroke pathogenesis. Astrocytes support neuronal viability by releasing mitochondrial particles via CD38 [164]. Moreover, their low-density lipoprotein receptor-related protein-1 (LRP1) reduces lactate production and ADP-ribosylation factor 1 (ARF1) lactylation. In stroke patients, high cerebrospinal fluid lactate levels correlate with astrocyte mitochondrial damage, highlighting the protective role of LRP1 in stroke through astrocyte-neuron crosstalk [165].

Research has shown that Nestorone, a progesterone analog, upregulates SIRT3 and restores the acetyl-superoxide dismutase 2 (Ac-SOD2)/superoxide dismutase 2 (SOD2) ratio by modulating the progesterone signaling pathway. This effectively improves mitochondrial metabolism and enhances oxidative protection, with even greater efficacy when combined with human amniotic fluid-derived stem cells (hAFScs) [166]. Ginsenoside Rb1 protects mitochondria by inhibiting NADH dehydrogenase in mitochondrial complex I, blocking reverse electron transport-derived ROS production, and inactivating astrocytes [167]. Additionally, cationic arginine-rich peptides (CARPs), which can target mitochondria, show promise in treating stroke [168].

In conclusion, the role of mitochondria in CVDs and stroke is complex and multilayered, involving energy metabolism, ROS production, mitochondrial quality control, and neuroinflammation. Targeting mitochondria offers a promising avenue for developing novel therapeutic strategies to reduce CVDs and stroke risk and improve outcomes.

### Metabolic disorders

#### Type 2 diabetes mellitus (T2DM)

Mitochondria are known to take a central stage in energy balance and cellular metabolism, which implicates their crucial role in metabolic disorders, especially in T2DM. Impaired insulin secretion from β-cells and IR are the typical pathophysiology features of T2DM, which have been both confirmed to be related to mitochondrial dysfunction [169, 170]. Downregulation of the mitochondrial fission protein Drp1 in INS1 cells leads to morphological changes, affecting mitochondrial membrane potential and ATP generation. This ultimately impairs glucose-stimulated insulin secretion (GSIS) [171]. Further studies show that Drp1 deficiency does not directly impede glucose-stimulated OXPHOS but instead compromises substrate delivery to mitochondria. This defect can be rescued by pyruvate supplementation [172]. Specifically, in the absence of Drp1, Mfn1/2 may be overexpressed to promote mitochondrial fusion, while the function of Fis1 may be suppressed. By modulating the insulin signaling pathways, such as the phosphatidylinositol-3-kinase (PI3K)/protein kinase B (Akt) pathway, the activity of PI3K is influenced, which in turn suppresses the phosphorylation of Akt. Additionally, dysfunction of glucose uptake and the glucose transporter 4 (GLUT4) reduces glucose utilization, worsening IR [173]. Beyond changes in mitochondrial morphology, other critical factors in β-cell dysfunction include increased ROS production, overexpression of uncoupling protein 2 (UCP2), and altered mitophagy [174].

IR, another hallmark of T2DM, occurs before reduced insulin secretion and is also linked to mitochondrial disturbances in skeletal muscle, adipose tissue, and the liver [170, 175]. Among them, skeletal muscle is the primary site of IR in T2DM patients. Multiple studies have reported abnormalities in mitochondrial number, density, and function in skeletal muscle using techniques such as transmission electron microscopy, OXPHOS enzymatic activity analysis, and mitochondrial substrate oxidation measurements [176–178]. Recent studies have further revealed the mechanism of mitochondrial engagement in IR. D. J. Fazakerley et al. showed that mitochondria-originated ROS impairs insulin-stimulated GLUT4 translocation, significantly resulting in IR [179]. Moreover, mitochondrial Ca^2+^ mishandling and disruption of ER–mitochondria interaction are other possible molecular mechanisms responsible for IR. Ca^2+^ mishandling leads to Ca^2+^ overaccumulation, which in mitochondria causes the opening of the mPTP and initiation of cell death, thereby affecting cellular energy supply and insulin signaling. The function of the mitochondrial calcium uniporter (MCU), which is responsible for Ca^2+^ transport in mitochondria, may be affected during IR. This results in decreased or dysregulated Ca^2+^ transport efficiency, thus exacerbating mitochondrial Ca^2+^ mishandling [180]. Disrupted ER-mitochondria interactions, such as reduced mitochondria-associated membranes, impair material exchange and information transfer between these organelles. This disruption enhances ER stress and mitochondrial stress, contributing to IR development [181].

Collectively, substantial evidence highlights the essential role of mitochondria in T2DM pathogenesis, from regulating insulin release and sensitivity to the onset of complications [182]. Notably, common T2DM treatments such as exercise, caloric restriction, metformin, and thiazolidinediones improve insulin sensitivity and mitochondrial function, benefiting T2DM patients [183–186]. Furthermore, mitochondrial-targeted therapies such as antioxidative agents and mitochondrial fission inhibitors lent unique insight into T2DM patients [183, 185]. Mitochondrial impairment is a common feature in various metabolic diseases, including obesity, liver diseases, CVDs, and more. These conditions are often accompanied by IR and an imbalance in energy metabolism [187]. Therefore, modulating Drp1 activity or Ca^2+^ homeostasis pathways may help alleviate symptoms of IR and other metabolic disorders. Antioxidants and mitochondrial fission inhibitors may serve as adjunctive therapeutic options.

#### Liver diseases

The liver is the major metabolic organ in the body and requires large amounts of energy to maintain its physiological functions. Mitochondria provide essential energy support to hepatocytes by producing ATP [188]. They are strongly associated with various liver diseases, including alcoholic liver disease (ALD), non-alcoholic fatty liver disease (NAFLD), and hepatic ischemia–reperfusion injury [189].

Alcohol exposure is a major cause of mitochondrial dysfunction in the liver. Chronic alcohol consumption inhibits the synthesis of mitochondrial respiratory complex proteins, reducing OXPHOS capacity, increasing ROS levels, and triggering oxidative stress [190]. Mitophagy combats ALD by removing damaged mitochondria [191]. Depletion of the Parkin gene accelerates alcohol-induced liver injury and fatty degeneration, likely due to impaired mitophagy in hepatocytes [192]. Impaired mitochondrial function is also a marker of disease progression in NAFLD. Hepatocytes from NAFLD patients show increased expression of proteins involved in mitochondrial fusion, and liver fat accumulation leads to systemic metabolic disorders [193]. It has been reported that BNIP3-mediated mitophagy is crucial for regulation hepatic lipid metabolism and may protect against NAFLD progression [194]. The mitochondrial deacetylase SIRT3 promotes BNIP3-mediated mitophagy against hepatocyte apoptosis via the extracellular signaling regulated kinase-cAMP-responsive component binding protein signaling pathway [194, 195]. In addition, IR is closely related to NAFLD and metabolic dysfunction-associated steatotic liver disease (MASLD). Impaired mitochondrial function leads to hepatic fat accumulation, defective insulin signaling, and dysregulated lipoprotein transport. Increased mtROS generation may contribute to IR and drive the progression of NASH and MASLD [196, 197].

During hepatic ischemia–reperfusion injury, mitophagy restores intracellular energy metabolism and reduces ROS and acidic metabolite accumulation by removing dysfunctional mitochondria. However, prolonged ischemia may impair autophagy, leading to ineffective clearance of damaged mitochondria [198].

Various methods of preventing and treating liver disease are being investigated. Treatments for mitochondrial related liver disease include the intake of low-calorie diets, antidiabetic drugs, bile acid modulators, antioxidants, mitochondrial uncouplers, and enhanced mitochondrial bioenergetics [189, 199–201]. Mitochondria-targeted antioxidants and naturally available herbs and polyphenols have also been reported to improve mitochondrial function and reduce oxidative stress [202–204].

In summary, mitochondria play a crucial role in metabolic disorders. Further in-depth studies on the specific mechanisms of mitochondrial action in metabolic disorders and the development of effective mitochondrial-targeted therapies are needed in the future.

### Cancer

**Fig. 3:**
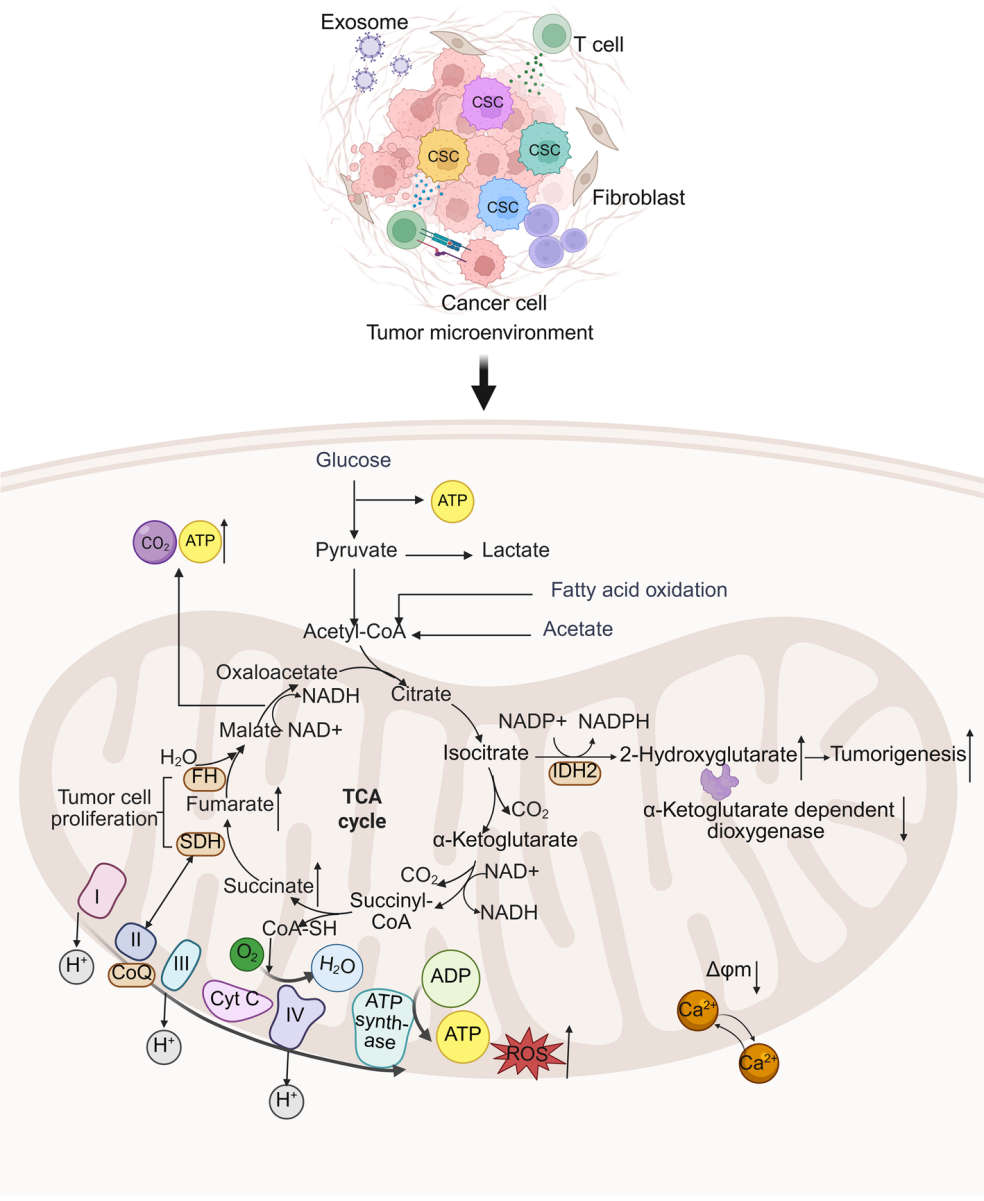
Mitochondrial bioenergetics and gene mutations in cancer. Glucose is shown entering the cell and being converted into pyruvate, which can then be further metabolized into lactate or enter TCA cycle. Pyruvate can also be used for fatty acid oxidation or converted into acetyl-CoA, which is a key molecule in the TCA cycle. In TCA cycle, acetyl-CoA into citrate, isocitrate, α-ketoglutarate, succinyl-CoA, succinate, fumarate, and malate, with the production of ATP, NADH, and FADH2. OXPHOS also results in the production of ROS, which can contribute to genomic instability and promote tumorigenesis. Certain metabolic enzymes and processes are altered in cancer cells to support their growth and survival. Gene mutations have been identified in FH, SDH (i.e., complex II), and IDH2 in cancer cells, and mtDNA mutations have been reported in the genes of complexes I, III, IV, and V

Mitochondria play fundamental roles in the bioenergetic metabolism and survival of cancer cells. Despite adequate oxygen supply, most cancer cells also produce energy through glycolysis, known as the Warburg effect [205]. Earlier studies suggested that cancer cells rely mainly on glycolytic pathways. More recent research has revealed the importance of mitochondrial function in tumorigenesis and progression [206]. We know that the mitochondrial genome includes mtDNA copies and the genes encoded by nDNA. Mutations in mtDNA have been identified in diverse cancers that alter mitochondrial metabolism, enhance tumorigenesis, and enable cancer cells to adapt to the changing environment [7, 207]. Additionally, mutations in nDNA genes such as succinate dehydrogenase (SDH), fumarate hydratase (FH), isocitrate dehydrogenase 2 (IDH2), and IDH3 in the mitochondrial tricarboxylic acid (TCA) cycle were also observed to result in increasing levels of succinate, fumarate, or 2-hydroxyglutarate [208–210]. These changes inhibit α-ketoglutarate-dependent dioxygenases and activate the NRF2 stress pathway, promoting tumorigenesis [211]. Altered mitochondrial metabolism also increases mtROS production and changes cellular redox status. It affects the activity of factors such as HIF-1α and activator protein 1 (AP-1), thereby altering gene expression and stimulating cancer cell proliferation [212, 213]. In addition, decreased mitochondrial membrane potential reduces mitochondrial Ca^2+^ uptake, limiting the activation of the mitochondrial apoptotic pathway and promoting cancer cell survival and proliferation (Fig. 3) [214].

Mitochondrial function is also critical in cancer stem cells (CSCs). CSCs not only have the ability to self-renew but also play a central role in tumor initiation, progression, spread, recurrence, and drug resistance [215]. There is no consistent view of the mitochondrial metabolic features of CSCs. Some studies suggest that CSCs rely preferentially on glycolysis, accompanied by low OXPHOS and high lactate production, while others point to OXPHOS as their main energy source [216, 217]. The role of glycolysis in maintaining the stemness of CSCs is widely recognized, particularly in a wide range of solid tumors. The phenomenon of mitochondrial fragmentation in CSCs also supports preferential glycolysis [218]. However, OXPHOS predominance is also observed in certain CSCs, such as those in pancreatic ductal adenocarcinoma and colon cancer [219, 220]. Overall, CSCs metabolic flexibility correlates with the hypoxic state of the tumor microenvironment (TME) and cellular subpopulation diversity. Targeting these processes requires precision to avoid side effects. Mitophagy is crucial for CSCs differentiation and maintains mitochondrial homeostasis alongside mitochondrial biogenesis, promoting CSCs plasticity to adapt to the TME [217, 221]. Mitophagy is one of the reasons for the failure of drug therapy in malignant tumors. For example, the resistance of colorectal CSCs to adriamycin is partly attributed to the BCL-2/BNIP3-like (BNIP3L)-mediated mitophagy process [222]. Thus, mitochondria in CSCs represent a challenging target for cancer therapy [223].

Tumor therapy is complicated not only by cancer cell heterogeneity but also by the complex TME, which includes tumor-associated macrophages (TAMs), dendritic cells (DCs), cancer-associated fibroblasts (CAFs), T cells, and other cells. TAMs undergo metabolic remodeling, including OXPHOS, fatty acid oxidation (FAO), and glycolysis, with upregulation of Arg1, promoting immunosuppressive pro-tumor signaling in the TME [224]. DCs are activated by the Toll-like receptor (TLR) 4 and are dependent on mitochondrial metabolic differentiation into type 1 conventional DC (cDC1) or type 2 conventional DC (cDC2) [225]. CAFs support tumor growth through metabolic reprogramming, mitochondrial translocation, and apoptosis resistance [226]. T cells within the TME rely on FAO to cope with nutritional stress, with CD8+ T cell function regulated by mitochondria [227]. Regulatory T cells in the TME depend on OXPHOS [228, 229]. The heterogeneity of TME requires different oxygenation, resulting in uneven mitochondrial distribution and function. The hypoxic regions in TME are associated with poor prognosis as it reduces mitochondrial metabolic activity and overexpression of related factors such as HIF-1, resulting in reduced energy production. Hypoxia induces mitochondria to adjust the number of electrons through the ETC, which leads to the excessive production of ROS, alters genomic stability and damages DNA repair pathways to promote the survival and proliferation of tumor cells [230].

All in all, targeting mitochondria for cancer therapy is a prospecting strategy through a deeper understanding of the specific mechanisms of mitochondria in cancer. Mitochondria are involved in cancer initiation, development, and progression, as illustrated in Fig. 3. Previous studies have shown that mtDNA mutations, OXPHOS dysfunction, ROS overproduction, and abnormal metabolite accumulation drive tumorigenesis [38]. Various mitochondrial inhibitors have been developed, with some in clinical trials. For example, IACS-010759 has been approved by the Food and Drug Administration (FDA) for use in phase I clinical trials in advanced cancers [231]. Meanwhile, there have been nanomaterial-based drug delivery targeted mitochondrial combination therapies showing long term anti-cancer potential [232, 233]. Until now, eliminating cancer cells without harming healthy cells has remained a formidable challenge.

### Infectious diseases

Mitochondria serve as a link between cellular metabolism and host defense mechanisms. Viruses, in particular, have evolved strategies to subvert the host's intracellular environment to favor their replication, often targeting mitochondrial functions and impacting metabolism and innate immune signaling. The coronavirus disease 2019 (COVID-19) is a deadly pandemic caused by severe acute respiratory syndrome coronavirus 2 (SARS-CoV-2), with a range of typical respiratory symptoms including fever, dry cough, fatigue and in severe cases dyspnea [234]. Emerging evidence suggests that mitochondrial dynamics interact with coronaviruses, playing a mechanistic role in COVID-19 development through anti-viral innate immune responses [14, 235, 236]. A study of peripheral blood monocytes from COVID-19 patients revealed metabolically impaired, with decreased respiration and glycolysis in monocytes from a functional and bioenergetic point of view, presenting as decreased basal and maximal respiration, lower spare respiratory capacity, and reduced proton leak. That leads to the redistribution of monocyte subsets: expansion of pro-inflammatory intermediate cells, along with the reduction of nonclassical monocytes, associated with the exaggerated inflammation in pneumonia [237].

Some studies have suggested that SARS-CoV-2 could influence mitochondrial function directly or indirectly in multiple mechanisms involving angiotensin-converting enzyme 2 (ACE-2), transmembrane serine protease 2 (TMPRSS2) [235], innate immunity functions [238]. ACE-2 serves as a primary entry point for SARS-CoV-2. ACE-2 serves as the primary entry point for SARS-CoV-2. Upon binding to ACE-2, the virus uses TMPRSS2 to facilitate fusion with the host cell membrane, enabling viral entry [239]. ACE-2 also regulates blood pressure and cardiovascular health by modulating the renin-angiotensin system. By downregulating ACE-2 activity, SARS-CoV-2 may disrupt this balance, leading to mitochondrial dysfunction, particularly in organs with high ACE-2 expression. These changes can result in reduced energy production, increased oxidative stress, and mitochondrial damage, contributing to multi-organ dysfunction in severe COVID-19 cases [235, 240]. SARS-CoV-2-induced inflammation may directly impair mitochondrial function, leading to elevated ROS production, which triggers additional pro-inflammatory cytokines and a cytokine storm, creating a vicious cycle [241]. The release of mtDNA is another inflammation mechanism correlated with the onset of multi-organ failure in acute respiratory distress patients [242]. Moreover, circulating mtDNA has been found to be an independent early risk factor for poor COVID-19 outcomes [243].

Beyond COVID-19, hepatitis C virus (HCV) initiates signaling pathways by blocking PRRs that recognize pathogen-associated molecular patterns (PAMP). This alters mitochondrial dynamics, promotes mitophagy, prevents premature cell death, and diminishes the interferon (IFN) response [244]. Hepatitis B virus (HBV) infection has been shown to impair mitochondrial function, which subsequently leads to disorders in lipid metabolism and abnormal mitochondrial function. These changes exacerbate the exhaustion of virus-specific CD8+ T cells, characterized by increased protein oxidation and DNA damage [245]. Research has demonstrated that decanoylcarnitine can enhance mitochondrial energy supply and promote mitochondrial homeostasis by activating the PPARα-carnitine palmitoyltransferase 1 A (CPT1A) signaling pathway. This ameliorates disruptions in hepatic lipid metabolism and mitochondrial function caused by HBV infection [246]. Furthermore, overexpression of inner membrane translocase complex (TIM) 22 and TIM29 has been observed to significantly inhibit HBV DNA and RNA levels, as well as the secretion of HBV surface antigen and E antigen [247]. The mitochondrial open reading frame of 12S rRNA-c (MOTS-c) serves as an antiviral molecule, inhibiting HBV replication by activating mitochondrial antiviral-signaling protein and its downstream pathways. Additionally, MOTS-c maintains mitochondrial homeostasis through myosin-9-actin molecular motor-mediated mitochondrial remodeling [248].

Altogether, mounting knowledge of mitochondrial mechanisms in the induction of COVID-19 has yielded insights into a potential alternative treatment apart from typical COVID-19 vaccines [249] against COVID-19 disease by selectively restoring mitochondrial function and strengthening mitochondrial biogenesis and quality control [14].

### Aging and mitochondrial decline

Aging is a multidimensional biological process characterized by the accumulation of cellular damage and the development of multiple diseases. It is closely linked to mitochondrial impairment, including mtDNA mutations, genomic instability, reduced mitophagy, and impaired biogenesis [5, 15]. As organisms age, mtDNA point mutations and deletions accumulate in somatic cells. These changes not only affect energy metabolism but also lead to oxidative damage. Oxidants produced by mitochondria are thought to be the main origin of oxidative damage, and SOD2 in the mitochondrial matrix plays a vital role in antioxidants by catalysing the conversion of superoxide anion to H_2_O_2_ [250].

Decreased mitochondrial ADP sensitivity during aging increases mitochondrial H_2_O_2_ emissions, causing age-related redox stress [251]. Reduced efficiency of OXPHOS leads to decreased ATP production and increased ROS generation. This excess ROS causes oxidative damage to mitochondrial DNA, proteins, and lipids, further impairing mitochondrial dynamics and hindering mitophagy. Ultimately, this leads to mitochondrial damage and reduced biogenesis [15, 252, 253]. More and more evidence suggests that MQC has a crucial role in maintaining mitochondrial homeostasis. MQC mechanisms include mitochondrial proteases, the mitochondrial unfolded protein response (UPR), mitochondria-derived vesicles (MDVs), and mitophagy [254, 255]. Mitochondrial proteases act as molecular scissors, meticulously trimming and degrading misfolded, damaged, or excess proteins within the mitochondrial matrix [256]. UPR is activated in response to stress conditions, such as increased levels of unfolded proteins within the mitochondria. The UPR is activated in response to stress, such as increased levels of unfolded proteins. Upon activation, it triggers a cascade of events aimed at restoring mitochondrial homeostasis, including enhancing the expression of genes encoding proteins involved in protein folding, degradation, and antioxidant defence [254]. Furthermore, MDV formed at the OMM, encapsulate and sequester unwanted or dysfunctional mitochondrial cargo, such as oxidized proteins and damaged membrane fragments. Subsequently, MDV fuse with lysosomes, allowing for the degradation of their contents within this cellular waste disposal system [255]. These mechanisms work together to maintain the quality and quantity of mitochondrial proteins and ensure proper mitochondrial function. However, excessive activation or inhibition of MQC can impair energy metabolism and mitochondrial function, accelerating aging [257]. The levels of nicotinamide adenine dinucleotide (NAD+) in the body decline with age, contributing to the progression of age-related diseases [258]. Recent studies show that nicotinamide mononucleotide (NMN) administration can restore NAD+ levels, prevent ovarian atrophy, and enhance oocyte quality and quantity. It also improves serum hormone secretion, antioxidant factors, and mitochondrial function while reducing inflammation. These findings suggest that restoring NAD+ levels may be an effective intervention against ovarian aging [259]. Additionally, myristoleic acid protects aged mice from muscle atrophy by reducing ROS accumulation and targeting peroxiredoxin 5, offering potential therapeutic insights for sarcopenia and healthy aging [260].

Notably, although mitochondria are closely linked to the aging process, the relationship between them is complex. Several studies have shown that mitochondrial dysfunction does not always directly lead to shortened lifespan. For example, increased oxidative stress in the nematode Caenorhabditis elegans does not necessarily shorten lifespan and may even activate the Krüppel-like factor-1 (KLF-1) transcription factor, which alters mitochondrial function to extend lifespan [261, 262]. In summary, aging is a complicated process that involves the decreased function of multiple organelles. Through intensive study of mitochondrial mechanisms, we can better understand the aging process and may discover new therapeutic strategies to slow down aging and improve the quality of life.

In conclusion, the role of mitochondria in disease is complex and far-reaching. Our understanding of mitochondrial function and its implications for health is still evolving. The potential to modulate mitochondrial function for therapeutic benefit is immense, offering new avenues for treatment and prevention of these prevalent conditions. Future research focus on elucidating the precise mechanisms by which mitochondria contribute to disease and on developing strategies to harness this knowledge for clinical benefit.

## Therapeutic strategies targeting mitochondria

**Fig. 4:**
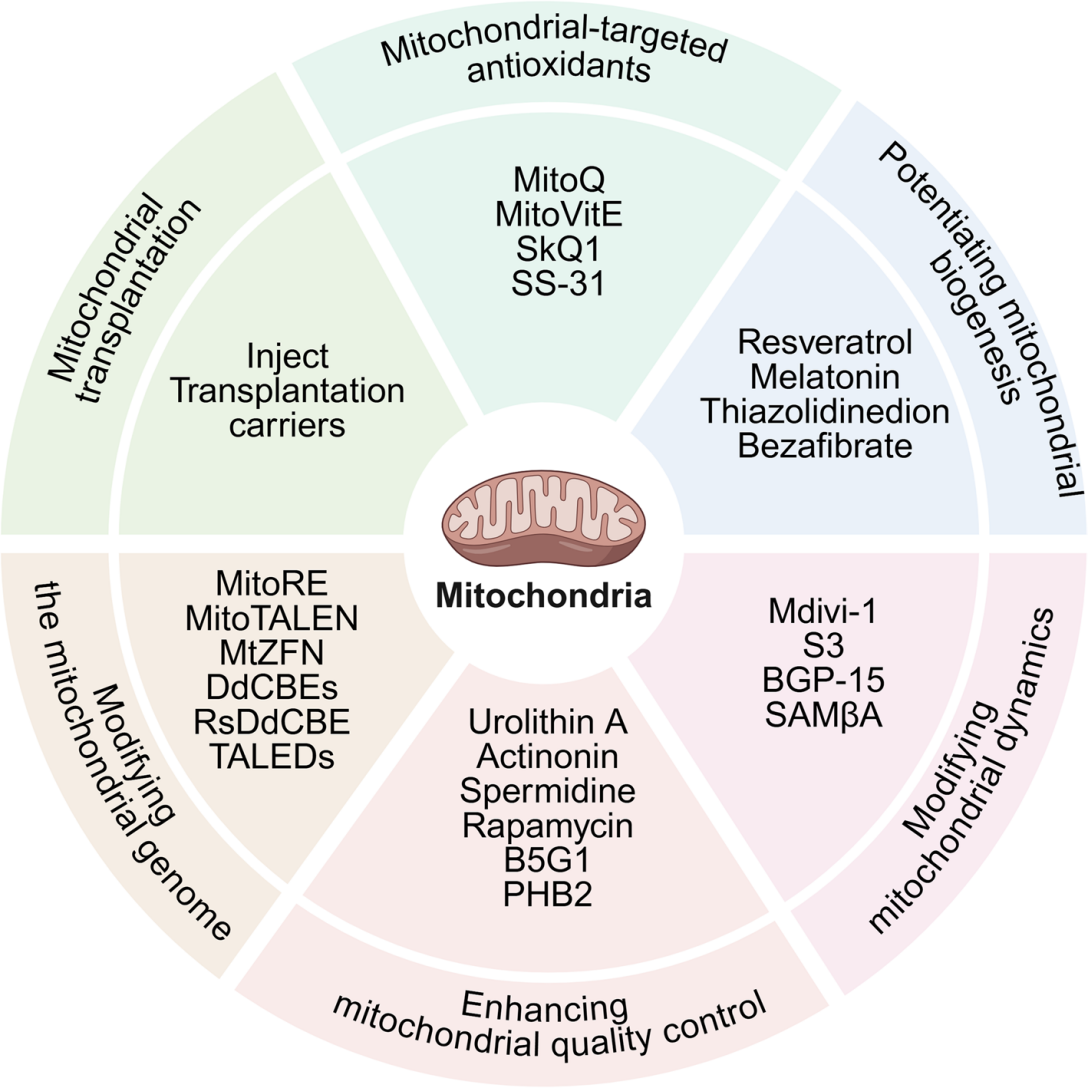
Some typical emerging therapeutic strategies for targeting the mitochondria. They fall into the following main categories: mitochondria-targeted antioxidants, modulating mitochondrial dynamics, enhancing mitochondrial quality control, potentiating mitochondrial biogenesis, modifying the mitochondrial genome editing and genetic therapy, and mitochondrial transplantation

Increasing evidence highlights the critical role of mitochondria in the development of multiple diseases, ranging from normal aging and cell senescence to common disorders. Intriguingly, similar patterns of mitochondrial damage contribute to pathology across various diseases, as discussed earlier. This underscores the potential of mitochondria-targeted therapies as an alternative approach for these prevalent conditions [38, 263, 264]. Accordingly, emerging therapeutic approaches fall into the following main categories: first, mitochondria-targeted antioxidants [16]; second, modulating mitochondrial dynamics and quality control [43, 265, 266]; third, modifying the mitochondrial genome editing and genetic therapy [267]; fourth, mitochondrial transplantation (MT) [21]. Notably, targeting one category of mitochondrial dysfunction often has a positive effect on others owing to the linkage of those damaged mitochondria. Many of these therapies have entered clinical trials, which are summarized in Table 2. Some typical emerging therapeutic strategies for targeting the mitochondria have been exhibited in Fig. 4.

**Table 2:** Interventions targeting mitochondria

Diseases	Intervention	Stage of development	Comment	NCT number
Mitochondrial disease	N-acetylcysteine	Phase I	Targeted antioxidant	NCT05241262
	KH176	Phase I	Intracellular redox modulating agent	NCT02544217
	Omaveloxolone	Phase II	NRF2 activator and NF-κB inhibitor	NCT02255422
	MitoQ	Phase II/III	Targeted antioxidant	NCT06191965
	ScAAV2-P1ND4v2	Phase I	Gene replacement therapy	NCT02161380
	Dichloroacetate	Phase III	Pyruvate dehydrogenase kinase inhibitor	NCT02616484
	MT1621	Phase III	Deoxynucleoside combination therapy	NCT04581733
	Vatiquinone	Phase II/III	Oxidative stress-protective agent	NCT04378075
Alzheimer’s disease	MitoQ	Not applicable	Targeted antioxidant	NCT03514875
	Centella asiatica product	Phase I	Herbal therapy	NCT05591027
Parkinson’s disease	Terazosin	Phase IV	α−1-adrenergic receptor antagonist	NCT05855577
	MitoQ	Phase II	Targeted antioxidant	NCT00329056
Amyotrophic lateral sclerosis	GM604	Phase II	Endogenous signal peptide for neuroprotection	NCT01854294
	Triheptanoin	Phase I/II	Replenish TCA intermediate	NCT03506425
Atherosclerosis	MitoQ	Not applicable	Targeted antioxidant	NCT03506633
	PB125	Not applicable	Dietary supplement	NCT05648630
Stroke	Idebenone	Phase IV	ATP production modulator and antioxidant	NCT05987397
Diabetes mellitus	Acipimox	Phase III	Antioxidant	NCT01816165
	Sodium-phenylbutyrate	Phase IV	Accelerate branched-chain amino acids oxidation	NCT05836350
	Dapagliflozin	Phase IV	Sodium-glucose cotransporter 2 inhibitor	NCT03919656
	Semaglutide + Dieting	Phase IV	Combination therapy	NCT04854083
	Isoleucine	Not applicable	Accelerate branched-chain amino acids oxidation	NCT04461236
	Metformin	Phase IV	Reduce blood sugar	NCT01813929
Diabetic nephropathy	Fenofibrate	Phase III	PPAR-α agonist	NCT03869931
Liver diseases	MitoQ	Phase II	Targeted antioxidant	NCT00433108
Systemic lupus erythematosus	N- Acetylcysteine	Phase II	Targeted antioxidant	NCT00775476
	Nicotinamide riboside	Phase I/II	NAD + boosting	NCT06032923
Multiple sclerosis	Dimethyl fumarate	Phase IV	Oxidative stress-protective agents	NCT02461069
	Ofatumumab	Not applicable	Anti-CD20 human monoclonal antibody	NCT05171972
	Idebenone	Phase I/II	Dietary supplement	NCT00950248
	Coconut oil + Epigallocatechin gallate	Phase II	Antioxidant	NCT03740295
	D-aspartate + IFN β−1a + Methylprednisolone	Phase II	Combination therapy	NCT03387046
Juvenile idiopathic arthritis	CoQ10	Phase II/III	Antioxidant	NCT05871086
Solid tumors	BGB-24714 ± Paclitaxel ± Carboplatin ± Docetaxel	Phase I	Combination therapy	NCT05381909
	Nivolumab/Pembrolizumab ± Metformin/Rosiglitazone	Phase II	Combination therapy	NCT04114136
Non-small cell lung cancer	Debio1143 + Avelumab	Phase I	Combination therapy	NCT03270176
	Radiation therapy + Carboplatin ± Paclitaxel ± Pemetrexed	Phase I	Combination therapy	NCT05136846
	Chemotherapy + Itraconazole	Phase II	Combination therapy	NCT03664115
Malignant central nervous system tumors	ONC206 ± Radiation therapy	Phase I	Combination therapy	NCT04732065
Advanced tumors	TB511 ± Pembrolizumab	Phase I/II	Combination therapy	NCT06400160
	TQB3909	Phase I	Targeting BCL-2 protein inhibitor	NCT04975204
	ME-344 + Bevacizumab	Early phase I	Combination therapy	NCT02806817
	Debio 1143 + Nivolumab	Phase I/II	Combination therapy	NCT04122625
	Gamitrinib	Phase I	Targeted Hsp90 inhibitor	NCT04827810
	Debio1143 + Avelumab	Phase I	Combination therapy	NCT03270176
	PANVAC-V + PANVAC-F + Sargramostim	Phase I/II	Combination therapy	NCT00088413
Colorectal cancer	CPI-613 + Fluorouracil	Phase I	Combination therapy	NCT02232152
	CPI-613 + mFOLFIRINOX	Phase I	Combination therapy	NCT05070104
Prostate cancer	GnRH agonist	Not applicable	Gonadotropin-releasing hormone agonist	NCT03867357
Symptomatic multiple myeloma	Fenofibrate	Phase II	Protein-folding inhibitor	NCT01965834
Ovarian cancer	Birinapant	Phase II	SMAC mimetic	NCT01681368
	Abemaciclib ± Letrozole ± LY3023414	Phase II	Combination therapy	NCT03675893
Fallopian tube cancer	Birinapant	Phase II	SMAC mimetic	NCT01681368
Non-hodgkin lymphoma/hodgkin lymphoma	CPI-613 + Bendamustine Hydrochloride	Phase I	Combination therapy	NCT02168140
Breast cancer	MitoQ	Not applicable	Targeted antioxidant	NCT05146843
	TQB3909	Phase I/II	Targeting BCL-2 protein inhibitor	NCT05775575
	Chemotherapy + Fasting-mimicking diet ± Metformin	Phase II	Combination therapy	NCT04248998
	ONC201	Phase II	Disruption of mitochondria	NCT03394027
	Metformin Hydrochloride + Doxycycline	Phase II	Combination therapy	NCT02874430
	Letrozol	Phase II	Aromatase inhibitor	NCT04568616
	Therapeutic estradiol + Exemestane	Not applicable	Combination therapy	NCT01385280
	Paclitaxel + Carboplatin + Cyclophosphamide/Doxorubicin ± Oncotherm EHY-2030	Phase II/III	Combination therapy	NCT05889390
	Quercetin + EGCG + Etformin + Zinc	Early phase I	Combination therapy	NCT05680662
Acute myeloid leukemia	CPI-613 + Cytarabine + Daunorubicin Hydrochloride	Phase I/II	Combination therapy	NCT02472626
	CPI-613 + Cytarabine + Mitoxantrone Hydrochloride	Phase I	Combination therapy	NCT02484391
	Pitavastatin + Venetoclax	Phase I	Combination therapy	NCT04512105
	Minnelide	Phase I	Hsp70 inhibitor	NCT03347994
Acute lymphoblastic leukemia	Cytarabine + Mitoxantrone ± Alvocidib	Phase II	Combination therapy	NCT02520011
	Levocarnitine	Early phase I	Levocarnitine supplement	NCT05501899
Chronic lymphocytic leukemia	Flavopiridol	Phase II	Stop the growth of cancer cells	NCT00098371
	Ibrutinib + Daratumumab	Phase II	Combination therapy	NCT03734198
Melanoma	Pembrolizumab ± Metformin	Phase I	Combination therapy	NCT03311308
	Pembrolizumab + Encorafenib + Binimetinib	Phase II	Combination therapy	NCT05304546
Pancreatic cancer	CPI-613 + mFOLFIRINOX	Phase I/II	Combination therapy	NCT03699319
Malignant glioma	Metformin + Radiation IMRT + Temozolomide	Phase II	Combination therapy	NCT04945148
Head and neck cancer	Cisplatin ± BMX-001	Phase II	Combination therapy	NCT06532279
Multiple myeloma	Cyclophosphamid + LCL161	Phase II	Combination therapy	NCT01955434
	Bortezomib + Vorinostat/Placebo to vorinostat	Phase III	Combination therapy	NCT00773747
Li Fraumeni syndrome	Metformin	Phase II	Reduce blood sugar	NCT01981525
Aging	MitoQ	Phase II	Targeted antioxidant	NCT06027554
	Metformin	Phase III	Reduce blood sugar	NCT04264897
	Dapagliflozin	Phase I/II	Sodium-glucose cotransporter 2 inhibitor	NCT04401904
	Nicotinamide riboside	Phase II	NAD + boosting	NCT02950441
	Rapamycin	Phase I	mTOR inhibitor	NCT01649960
	Resveratrol	Phase II	Antioxidant	NCT02123121
COVID-19	Ferinject	Phase IV	Iron supplement	NCT05708170
	Arginine hydrochloride	Phase II	Protein supplement	NCT05855330
	Glycine + N-acetylcysteine	Early phase I	Targeted antioxidant	NCT04703036
	Isotretinoin + Standard treatment	Phase III	Combination therapy	NCT04353180
	Ubiquinol	Not applicable	Antioxidant	NCT05178225
	MitoQ/MitoQ-MES	Phase II	Targeted antioxidant	NCT05886816
	Trimetazidine	Phase II	KAT competitive inhibitor	NCT04760821
	Nicotinamide riboside + Mind–body reprocessing therapy	Phase II	Combination therapy	NCT05703074

+ : combination; ± : with or without;/: or

### Mitochondria-targeted antioxidants

MtROS, a primary type of ROS produced by the mitochondrial respiratory chain, not only participate in multiple pathologies directly but also regulate ROS production from non-mitochondrial sources [268], occurring as a secondary contributor to the pathological progression of many diseases including CVDs, T2DM and neurodegenerative diseases [8]. Thus, mitochondria-targeted antioxidant therapies, which ameliorate oxidative stress caused by excessive ROS production in mitochondria, hold promise as a selective treatment for various pathologic conditions.

Conventional antioxidant therapies such as resveratrol, vitamin C, vitamin E, and N-acetylcysteine have displayed therapeutic potential in preclinical studies of many diseases [8, 269]. However, clinical trial results are not so satisfactory due to difficulties in targeted delivery to diseased tissues and the potential disruption of ROS-associated physiological signaling pathways [270]. In contrast, mitochondria-targeted antioxidants can penetrate the mitochondrial phospholipid bilayer and accumulate within mitochondria, effectively reducing ROS at their primary source [19, 271]. This targeted approach minimizes off-target effects and enhances therapeutic efficacy. Common mitochondria-targeted antioxidants include Mitoquinone (MitoQ), MitovitE, Visomitin (SkQ1), and Szeto-Schiller (SS)−31 [272]. These compounds are designed to penetrate the mitochondrial phospholipid bilayer and accumulate within the mitochondria, effectively reducing ROS levels.

MitoQ, a derivative of ubiquinone attached to a lipophilic cation triphenylphosphonium (TPP), is the most characterized mitochondria-targeted antioxidant so far [273]. TPP, a well-known mitochondrial targeting moiety, enables MitoQ to accumulate up to 100–1000 times more within mitochondria due to the large membrane potential. This reduces the production of lipid peroxyl radicals and attenuates peroxynitrite-mediated oxidative damage. Since its discovery in the 1990 s, efficacious outcome of MitoQ in ameliorating oxidative stress has been reported in the preclinical mammalian animal model studies of several diseases, including neurodegenerative disease [271], diabetic kidney disease [274], inflammatory bowel disease [275], lupus [276], and cancer [277]. The protective effects and safety of MitoQ have been proved in Phase II clinical trials in liver disease [278]. However, MitoQ's efficacy in altering disease progression in PD patients over 12 months was limited, likely due to insufficient brain penetration and severe dopaminergic deficiency [279]. In healthy older adults, MitoQ supplementation improved vascular endothelial function, reduced age-related aortic stiffening, and decreased plasma oxidized LDL levels [280]. Ongoing clinical trials continue to explore MitoQ's potential therapeutic applications. While not as widely tested as MitoQ, SkQ1, a derivative of plastoquinone conjugated to TPP, has received commercial approval as a droplet formulation (called Visomitins) for dry eye symptoms in Russia [281].

SS-31, a peptide belonging to specific targeting antioxidant peptides, is another vital type of mitochondria-targeted antioxidant [282]. Its alternating aromatic-cationic structure allows it to concentrate in the IMM independently of the mitochondrial membrane potential. SS-31 inhibits mtROS production and decreases oxidative damage by interacting with mitochondrial cardiolipin, protecting the structure of mitochondrial cristae. Researches have shown the therapeutic potential of SS-31 in extensive animal models of many highly complex diseases, especially age-associated diseases such as type 2 diabetes, age-related heart dysfunction and neurodegenerative diseases [283–285]. Meanwhile, SS-31 has been suggested to target protein anabolism/catabolism for the prevention of cancer cachexia by modulating skeletal muscle and liver metabolome in C26-bearing mice [286]. Plenty of clinical trials have been performed or are undergoing to test the efficacy and safety of SS-31 in a wide range of human diseases such as heart failure, primary mitochondrial myopathy, and other mitochondrial diseases [282, 287]. Collectively, these studies suggest that SS-31 could be a cornerstone of future multimodal therapies.

Despite the promising potential of mitochondria-targeted antioxidants, several challenges remain. Targeted delivery to specific tissues and cells, especially in the brain, is a significant hurdle [288]. Additionally, the long-term effects of these antioxidants on mitochondrial function and overall cellular health need further investigation. Future research should focus on optimizing delivery mechanisms, enhancing tissue specificity, and evaluating long-term safety and efficacy in diverse patient populations.

### Modulating mitochondrial dynamics and quality control

#### Modulating mitochondrial dynamics

In general, increased fission or decreased fusion activity is a vital component of disease pathophysiology, especially in neurological disorders, cardiac dysfunction and different types of cancer. Hence, re-establish balanced fusion and division by using small molecules or genetic approaches appears to be a viable approach.

Mitochondrial division inhibitor-1 (Mdivi-1) is a well-characterized drug that specifically inhibits Drp1 activity, affecting mitochondrial dynamics. Since its discovery in 2008, the beneficial effects of Mdivi-1 have been well-confirmed in diseases characterized by excessive mitochondrial fragmentation. In mouse models of PD and AD, Mdivi-1 treatment reduced mitochondrial fragmentation and neurodegeneration, improving behavioral outcomes [289, 290]. In pre-diabetic rats, Mdivi-1 significantly attenuated cardiac mtROS production and improved left ventricular function [291]. Drp1 siRNA knockdown exhibits similar effects. In human cancer, Mdivi-1 impaired cell proliferation by decreasing oxidative metabolism in human breast and lung cancer cells [292, 293]. Additionally, Mdivi-1 has been shown to cooperate with cisplatin to induce apoptosis in cholangiocarcinoma, breast carcinoma, and ovarian cancer cells [294–296]. Given the temporal correlation between disease progression and alterations in mitochondrial morphology, determining the optimal temporal window for Mdivi-1 application is crucial. P110 is another selective peptide inhibitor of Drp1, disrupting mitochondrial division by blocking the interplay between Drp1 and Fis1 [297]. Like Mdivi-1, P110 has shown therapeutic promise in models of neurodegenerative diseases [298]. However, more extensive clinical trials are required to validate its efficacy and safety. Therapeutic modulation of mitochondrial dynamics can lead to various side effects, depending on the specific drug and its mechanism of action [299].

In addition to these compounds that inhibit fission factors, enhancing mitochondrial fusion activity is another therapeutic approach, rebalancing aberrant mitochondrial dynamics. 15-oxospiramilactone (S3) is a chemical inhibitor of the mammalian deubiquitylase USP30, which increases the ubiquitylation of Mfn1 and Mfn2, promoting the elongation of the mitochondrial network [300]. In *Mfn1*^*−/−*^* or Mfn2*^*−/−*^ mouse embryonic fibroblast cells, S3 treatment restored mitochondrial fusion activity, ATP production and oxidative respiration, suggesting the potential therapeutic applications of S3 [301]. BGP-15, a hydroxylamine derivative, could promote mitochondrial fusion by activating OPA1. In murine and rabbit models of atherosclerotic cardiomyopathy, BGP-15 alleviates symptoms of heart failure and improves diastolic function [302]. In addition, SAMβA, another novel small peptide, prevents the accumulation of fragmented mitochondria by selectively inhibiting the interaction of the βII isoform of protein kinase C (βIIPKC) with Mfn1, which re-establishes morphology and function of mitochondrial and improves heart failure outcome in a rat model for heart failure [303]. Further focus on re-establishing balanced fusion and division will be essential for the development of effective therapeutics.

#### Enhancing mitochondrial quality control

MQC systems are crucial for maintaining mitochondrial function. Mitophagy, a key component of autophagy, eliminates dysfunctional mitochondria and is associated with various pathological conditions, particularly carcinogenesis [304] and neurodegenerative diseases [305]. Pharmacological modulation of mitophagy, either by activation or inhibition, is a topic of considerable interest.

Enhancing mitophagy to facilitate the removal of old or damaged mitochondria has shown ideal amelioration of symptoms in many mouse models of neurodegenerative disease [252]. Several natural compounds have been identified as novel mitophagy modulators and demonstrate protective effects against Aβ-induced mitochondrial and synaptic toxicities in animal models of AD. Examples include urolithin A [306], actinonin, and polyamine spermidine [109]. In addition, the safety and tolerability of urolithin A and polyamine spermidine have been observed in human clinical trials [307]. Improved mitochondrial and cellular health was reported in healthy, sedentary elderly individuals following 4 weeks of supplementation with urolithin A at doses of 500 mg and 1,000 mg [308]. Mechanistically, spermidine induces mitophagy by inhibiting acetyltransferase EP300 [309] and participating in the ataxia telangiectasia mutated (ATM)-mediated activation of the PINK1/Parkin pathway [310]. As mentioned above, the PINK1/Parkin axis is the most studied ubiquitin-dependent mitophagy pathway. Aside from the aforementioned natural compounds mitophagy inducer, small-molecule activators of PINK1 and Parkin, as well as inhibitors of USP30 (an upstream deubiquitinating enzyme that limits Parkin action and mitophagy), offer another approach to enhancing mitophagy [311, 312].

Rapamycin, a pharmacological inhibitor of the mechanistic target of rapamycin (mTOR), activates autophagy/mitophagy in a PINK1/Parkin-dependent manner [313]. In the AD mouse hippocampus, the impairment of cognitive ability and synaptic plasticity was significantly alleviated by rapamycin, which was attributed to the clearing of damaged mitochondria and reduced oxidative damage [314]. Meanwhile, rapamycin and its derivatives have also been explored in preclinical cancer treatment studies [315–317].

The role of mitophagy in cancer progression is still controversial, depending on the type and stage of cancer [318]. Thus, instead of constitutive activating mitophagy, careful modulation of mitophagy appears to be an appropriate choice in cancer treatment. Both inhibitors and activators of mitophagy have been demonstrated to take effect in anti-cancer therapy. However, mitophagy inhibition seems to be more efficient in many different human cancers. Blocking PINK1/Parkin-dependent mitophagy sensitizes multidrug-resistant liver cancer cells to B5G1, a new derivative of betulinic acid [319]. Prohibitin 2 (PHB2) is a novel inner membrane mitophagy receptor, mediating PINK1/Parkin-mediated mitophagy [320]. A recent study found that inhibiting PHB2-mediated mitophagy using PHB ligands (e.g., FL3) or shPHB2 effectively blocked cancer cell proliferation, revealing that targeting PHB2-mediated mitophagy is a promising strategy for cancer therapy [321].

#### Potentiating mitochondrial biogenesis

Increasing evidence reveals that dysregulated mitochondrial biogenesis occurs in the pathogenesis of several diseases, highlighting mitochondrial biogenesis activators as potential therapeutic approaches [69]. PGC-1α is a central regulator of transcriptionally governing mitochondrial biogenesis by activating several transcription factors such as NRF1, NRF2, and PPARγ [322]. Natural compounds like astaxanthin [323] and resveratrol [324] have been shown to interact with PGC-1α pathways, modulating downstream transcription factors and stimulating mitochondrial biogenesis. Melatonin, a hormone related to biological rhythms, enhances mitochondrial biogenesis and provides protective effects by targeting the AMPK/PGC-1α pathway in a range of disease models, including diabetic cardiomyopathy [325], liver fibrosis [326], T2DM [327], and AD [328]. In animal models of AD, treatments of pioglitazone reduced amyloid deposition and neuroinflammation, ameliorating cognitive deficits [329]. Moreover, clinical studies of PPARγ agonists have been performed in a wide range of health conditions [330]. However, inducing mitophagy and mitochondrial biogenesis can have unintended consequences. Excessive activation of mitophagy may deplete functional mitochondria, impairing cellular energy metabolism and causing fatigue or muscle weakness [331, 332]. Long-term use of these agents may also lead to cumulative toxicity, particularly in tissues with high mitochondrial density, such as the heart and liver.

### Modifying mitochondrial genome editing and genetic therapy

Mitochondria possess their own genome, the mtDNA. However, because mitochondria are located in the cytoplasm, the guide RNA of many existing genome-editing tools (e.g., the CRISPR/Cas system) cannot directly access mtDNA. This limitation restricts the application of these tools in editing the mitochondrial genome [333, 334]. Additionally, mtDNA sequences in mammalian cells are relatively stable and difficult to alter. Therefore, developing efficient methods to remove harmful mtDNA sequences or to transfer mitochondria with specific mtDNA sequences into cells is a critical step [335]. Heterogeneity in the intracellular distribution of the mitochondrial genome further complicates editing efforts [336]. Despite the many challenges, scientists continue to explore and improve techniques for modifying the mitochondrial genome.

To overcome the limitations of the CRISPR/Cas system in directly editing the mitochondrial genome, researchers have developed nuclease-based methods such as mitochondrial targeted restriction endonuclease (mitoRE) [337], transcriptional activator-like effector nuclease (mitoTALEN) [338], and zinc finger nuclease (mtZFN) [339]. These nucleases can be fused to mitochondrial targeted signal sequences, triggering double-stranded breaks in mtDNA and thus editing. However, this approach is prone to triggering heterogeneity in the mitochondrial genome and lacks suitable selection markers [340]. In recent years, a novel bacterial deaminase, DddA, has been discovered that directly modifies bases on double-stranded DNA. Based on this discovery, DddA-derived cytosine base editors (DdCBEs) were developed. These editors can convert base C to T in mtDNA, inducing point mutations without cutting the DNA [341]. Experiments with DdCBEs in vivo in mice and in human embryos demonstrate the feasibility of modifying the mitochondrial genome [342]. However, this approach is largely restricted to C to T conversions and causes extensive off-target effects [343]. To address these limitations, recent studies have successfully achieved in vivo mtDNA editing by delivering DdCBEs to the mouse heart using adeno-associated viral (AAV) vectors. This provides a new strategy for gene correction in mitochondrial diseases [344]. Further study enhanced mtDNA editing efficiency and reduced off-targeting editing in mouse embryos by fusing nuclear export signals with DdCBEs, while co-injection with TALEN improved editing effects [345]. Additionally, the identification of DddA homologs from Simiaoa sunii and their derived editors (DdCBE_Ss) has expanded the editing toolbox, improving editor activity and DNA sequence compatibility [346]. The researchers also identified a new DddAtox homolog from Ruminococcus sp. AF17-6 (RsDddA) through protein engineering. They demonstrated that the RsDddA-derived cytosine base editor (RsDdCBE) overcomes sequence context constraints of DddA, improving editing efficiency and targeting range [347]. These findings demonstrate the potential of DdCBEs in correcting pathogenic mtDNA mutations in human cells and provide scientific evidence for the future treatment of mitochondrial diseases, such as LHON, MELAS, and Leigh syndrome. Optimizing DdCBE tools to improve editing efficiency and reduce off-target effects is crucial for these applications.

Another emerging gene editing technology, transcription-activator-like effector (TALE)-linked deaminases (TALEDs), enables base A to G conversion in mtDNA, greatly expanding the scope of mitochondrial genome editing [348, 349]. TALEDs consist of TALE DNA-binding arrays, DddA variants, and TadA proteins that work together to achieve precise editing of the mitochondrial genome [350]. The introduction of overlapping TALE in the constructed TALEDs tiling array may reduce or eliminate non-specific editing that may be triggered by TALADs. Nevertheless, TALED systems may still be at risk of off-target editing [348]. As gene-editing technologies continue to develop, modifying the mitochondrial genome will become a valuable tool for treating mitochondrial genetic diseases. These tools hold great potential for personalized gene therapy for mitochondrial genetic diseases.

### Mitochondrial transplantation

In recent years, conventional treatments have had limited efficacy in curing these diseases caused by mitochondria. Mitochondrial transplantation (MT) has emerged as an innovative treatment by delivering healthy mitochondria to damaged cells or tissues. This approach replaces defective mitochondria and restores their function [5, 21]. Studies have demonstrated positive results of MT in various disease models, including central nervous system diseases [351], CVDs [352], inflammatory diseases [353], cancer [354, 355], and renal and lung injuries [20, 356].

MT can be classified into two main approaches. The direct transplantation of mitochondria into recipient cells via blood injection or in situ injection [357]. The other is the indirect delivery of mitochondria into damaged tissues or cells through the transplantation of other carriers carrying mitochondria, such as cells (haematopoietic stem cells, mesenchymal stem cells (MSCs), etc.) [358]. Despite potential variations in its effectiveness across different tissues, MT consistently rejuvenates ATP production and reduces ROS release from compromised mitochondria [5]. Additionally, MSCs inhibit the activation of the TLR signaling pathway in macrophages by transferring microRNA, thereby reducing inflammation and promoting a favorable environment for tissue repair and regeneration [353]. Recent investigations have revealed that exogenous mitochondria are efficiently internalized by various neural cells, including neurons, astrocytes, and microglia. MT has emerged as a promising strategy for protecting against traumatic brain injury by enhancing neuronal survival, restoring Tom20 expression and JNK phosphorylation, and increasing brain-derived neurotrophic factor (BDNF) levels in reactive astrocytes [359]. Furthermore, MT has been proposed to mitigate ischemia–reperfusion injury in the myocardium. A CSTSMLKAC (PEP)-TPP-mitochondria complex has been shown to promote mitochondrial uptake by cardiomyocytes and facilitate mitochondrial transfer from endothelial cells to cardiomyocytes. This reduces cell apoptosis, macrophage infiltration, and pro-inflammatory responses, thereby alleviating ischemia–reperfusion injury [360]. Research has explored the therapeutic potential of MT for idiopathic inflammatory myopathies. Studies involving in vitro and in vivo models, as well as the first-in-human clinical trial, have assessed the efficacy and safety of mitochondria isolated from human umbilical cord mesenchymal cells [361]. Additionally, inter-cellular mitochondrial transfer has been investigated as a novel approach in organ medicine. This method enhances T cell anti-tumor responses and extends animal survival times, laying the groundwork for next-generation cell therapies [362]. Interestingly, gender differences may affect MT efficiency. For example, healthy mitochondria from female sources may be more effective in delaying tumor growth and metastasis [363]. In another innovative approach, high-quality mitochondrial microfactories were constructed by anchoring Prussian blue nanozymes onto MSCs. This method facilitates effective MT for treating spinal cord injuries [364]. Moreover, in the context of liver transplantation, biomarkers of mitochondrial damage have been identified as potential predictors of graft loss, biliary complications, and renal failure [365]. Collectively, these findings highlight the broad therapeutic potential of MT across various medical applications.

Despite its potential, prolonged presence of exogenous mitochondria in vivo may trigger inflammatory responses. During depolarization, mitochondria may expose cardiolipin, enhancing NLRP3 activation and immune responses via TLR9 [366]. Therefore, developing efficient mitochondrial delivery systems to promote rapid cellular internalization is crucial. Notably, the clinical translation of MT remains faced with challenges, including the isolation of mitochondria, transplant rejection, blood–brain barrier, and ethical issues [367–369]. Furthermore, the choice of mitochondrial source, whether autologous, allogeneic or heterologous, is influenced by immunocompatibility and ethical and clinical application requirements. Autologous mitochondria are preferred due to their high biocompatibility but may be limited by underlying systemic or congenital mitochondrial diseases [370]. Allogeneic mitochondria offer a practical alternative, while heterologous mitochondria are mainly restricted to experimental studies due to immunogenicity risks and ethical concerns [371, 372]. Overall, as an emerging therapeutic strategy, MT offers new possibilities for treating a wide range of diseases by improving mitochondrial function in damaged cells. Although challenges remain, further research and technological advancements are expected to make MT an effective treatment for mitochondrial damage-related diseases.

## Challenges and future directions

### Research gaps in mitochondrial biology and therapy

Mitochondrial biology is a complex and rapidly evolving field, with significant gaps in our understanding of the organelle's role in disease pathogenesis and therapeutic potential. One primary challenge is the genetic complexity of mitochondria. Mitochondria possess their own DNA and are influenced by nuclear genes, resulting in dual-genome control that complicates the development of targeted therapies [373]. Moreover, technical challenges exist in delivering mitochondria to target cells with precision and efficiency. Improper mitochondrial integration can disrupt cellular functions or trigger inflammatory responses. Furthermore, the potential for immune responses is a major concern. The recipient's immune system may recognize transferred mitochondria as foreign entities, leading to immune rejection [374]. Patient variability is another critical factor affecting the efficacy of mitochondrial therapy. Individuals with mitochondrial diseases may respond differently to treatments due to the genetic and phenotypic heterogeneity of these conditions [375]. Factors such as age, gender, genetic background, and underlying health conditions can influence drug metabolism and response. For example, elderly patients may have reduced liver and kidney function, leading to slower drug clearance and increased risk of adverse effects. Gender differences can also affect drug pharmacokinetics and pharmacodynamics. For instance, hormonal variations in women can alter drug metabolism rates [376]. Genetic polymorphisms in drug-metabolizing enzymes can further contribute to inter-individual differences in drug response, necessitating personalized dosing strategies [377, 378]. Current therapeutic approaches are mostly limited to symptomatic treatments and supportive measures, such as the use of vitamins, cofactors, and antioxidants. Personalized medicine, leveraging genetic and phenotypic data, can optimize dosing regimens and minimize adverse reactions [379]. Additionally, comprehensive clinical trials that account for patient variability will be crucial in establishing the safety and efficacy profiles of these therapies [380].

### Potential solutions and innovative approaches

Innovative approaches in mitochondrial therapy are emerging, focusing on modulating mitochondrial dynamics and mitochondrial genetic therapy. One promising strategy involves using pharmacological agents that directly target these aspects of mitochondrial function. For example, the ketogenic diet has been shown to provide alternative energy to various organs and has potential benefits in conditions such as epilepsy [381], CVDs [382], and neurodegenerative diseases by increasing ketolysis and triggering an adaptive cellular response [383]. Additionally, the development of mitochondria-targeted nanomaterials offers a new avenue for drug delivery and disease management, with potential applications in cancer treatment, probes, and imaging [384, 385]. These nanomaterials can exploit the unique properties of mitochondria. For example, fluorinated amphiphilic molecules can enhance cellular uptake and mitochondrial localization, presenting a potential avenue for cancer treatment [386]. Luminoptogenetics gene therapy involves synthesizing a blue light-gated channelrhodopsin in the IMM and co-expressing a blue bioluminescence-emitting nanoluciferase in the cytosol. These components are selectively delivered to cancer cells [387].

### Future clinical and translational applications

The future of mitochondrial therapy lies in translating these innovative approaches into clinical applications. The use of targeted mitochondrial nanomaterials in biomedicine is particularly promising, with potential for diagnostic and therapeutic advancements [388]. Integrating nanotechnology with mitochondrial therapy may lead to breakthroughs in managing diseases associated with mitochondrial impairment. This offers hope for improved patient outcomes and quality of life. The combination of mitochondrial targeting polymers and photothermal effects can enhance cancer treatment efficacy, especially when used with immunomodulators and programmed death-ligand 1 (PD-L1) inhibitors [389]. As our understanding of mitochondrial biology expands, we can expect the development of more targeted and personalized treatments that address the underlying causes of mitochondrial dysfunction.

## Conclusion

In this review, we summarized the physiological functions of mitochondria, the roles of mitochondria in the process of several diseases, and emerging therapy strategies targeting mitochondria. Mitochondria are responsible not only for energy supply but also for processes such as signal transduction and cell death. The dynamic processes of mitochondrial fission and fusion regulate mitochondrial morphology, distribution, and quantity. These processes are vital for ensuring optimal mitochondrial function and cellular homeostasis. Additionally, mitophagy maintains mitochondrial quality control by eliminating damaged mitochondria, preventing the propagation of dysfunction. Although many core functions of mitochondria have been elucidated, many mechanisms remain unclear. For instance, there is still limited knowledge about how mitochondria precisely regulate their internal environment, particularly the dynamic balance between redox state, ion concentrations (e.g., Ca^2+^, H^+^), and cytoplasm. Using Ca^2+^ as an example, as an intracellular second messenger, mitochondria control the transmembrane flow of Ca^2+^ through its unique MCU and other related proteins, but the control mechanism and its relationship with disease remain to be explored [390, 391].

Increasing evidence highlights the role of mitochondrial mechanisms in common diseases, including mitochondrial disorders, neurodegenerative diseases like Alzheimer's and Parkinson's, CVDs and stroke, metabolic disorders such as T2DM, cancer, infectious diseases like COVID-19, aging and mitochondrial decline. In neurodegenerative diseases, although the mechanism of autophagy in clearing damaged mitochondria is understood, delivering therapeutic molecules across the blood–brain barrier without harming healthy neurons remains a pressing challenge [392]. Besides, the regulation of fatty acid metabolism by mitochondria and the formation mechanism of IR in metabolic diseases such as diabetes mellitus and liver diseases are the hot topics of current research [175, 393]. In the field of tumor therapy, mitochondria show promising therapeutic perspectives by disrupting mitochondrial energy metabolism, inducing mitochondria-mediated apoptosis or enhancing the immunogenicity of tumor cells [38]. However, accurate identification and intervention of different types of tumor cells while avoiding false damage to normal cells has remained a formidable challenge. Future studies are needed to better understand mitochondrial heterogeneity and specificity in various diseases to develop more precise treatments. For example, modification of specific disease-associated mitochondrial proteins or editing of mtDNA may provide new ideas for therapy.

A great deal of therapeutics targeting mitochondria have proved to bring renewed hope for the treatment of important pathologies. Approaches such as mitochondria-targeted antioxidants, modulating mitochondrial dynamics and MQC, mitochondrial genome editing, genetic therapy, and MT help maintain mitochondrial fitness. There are still other viable pathways targeting mitochondria such as manipulating mitochondrial signaling, harnessing the mitochondrial drug delivery system [394], and improving mitochondrial metabolism of the natural killer cells to recover natural killer cell-mediated surveillance in TME [395]. With advances in gene-editing techniques, personalized therapies for individual-specific mitochondrial gene mutations may become feasible. We anticipate being able to modify the mitochondrial genome more precisely, offering new strategies for treating mitochondrial genetic diseases. However, several challenges remain, including optimizing delivery mechanisms, minimizing off-target effects, and ensuring long-term safety and efficacy. In addition, mtDNA's genetic mode differs from conventional nDNA's, so the ethical issues involved in modifying mitochondrial genes cannot be ignored and need the joint discussion of the social, legal and scientific communities. Undoubtedly, we are approaching the new age of mitochondrial medicine. However, translating promising mitochondrial drugs from animal models to clinical use requires more basic research and clinical studies.

In conclusion, the normal function of mitochondria is of great significance in maintaining cellular homeostasis and preventing the occurrence of diseases. Challenges in mitochondrial research include understanding complex interactions between mitochondria and other cellular components and developing safe and effective therapeutic interventions. Future research should focus on addressing these challenges and exploring new therapeutic avenues in this vital field.

## Data Availability

Not applicable.

## Abbreviations

AAV: Adeno-associated viral
ACE-2: Angiotensin-converting enzyme 2
Ac-SOD2: Acetyl-superoxide dismutase 2
AD: Alzheimer’s disease
Akt: Protein kinase B
ALD: Alcoholic liver disease
ALS: Amyotrophic lateral sclerosis
AMPK: AMP-activated protein kinase
AP-1: Activator protein 1
APAF1: Apoptotic peptidase activating factor 1
ARF1: ADP-ribosylation factor 1
α-syn: α-Synuclein protein
ATF-2: Activating transcription factor-2
ATG9 A: Autophagy essential proteins autophagy-related 9A
ATM: Ataxia telangiectasia mutated
ATP: Adenosine triphosphate
Aβ: Amyloid β
BNIP3: Adenovirus E1B 19 kDa interacting protein 3
BNIP3L: BNIP3-like
BOK: B cell lymphoma 2 ovarian killer
BAX: BCL-2 associated protein X
BAK: BCL-2 antagonist killer
BCL-2: B cell lymphoma-2
BCL2L13: BCL-2-like protein 13
BDNF: Brain-derived neurotrophic factor
BH3: BCL-2 homology 3
βIIPKC: βII isoform of protein kinase C
cAMP: Cyclic adenosine monophosphate
CAMK: Calmodulin-dependent protein kinase
CAFs: Cancer-associated fibroblasts
CARPs: Cationic arginine-rich peptides
cDC1: Type 1 conventional DC
cDC2: Type 2 conventional DC
CK2: Casein kinase 2
COVID-19: Coronavirus disease 2019
COX: Cyclo-oxygenase
CPT1 A: Carnitine palmitoyltransferase 1A
CREB: CAMP response element-binding protein
CSCs: Cancer stem cells
Cyt: Cytochrome
CVDs: Cardiovascular diseases
DAMPs: Damage-associated molecular patterns
DCs: Dendritic cells
DdCBEs: DddA-derived cytosine base editors
Drp1: Dynamin-related protein 1
ΔΨm: Mitochondrial membrane potential
ECs: Endothelial cells
eNOS: Endothelial NO synthase
ER: Endoplasmic reticulum
ERAD: Endoplasmic reticulum-associated degradation
ERRα: Estrogen-related receptor α
ETC: Electron transport chain
FADH2: Flavin adenine dinucleotide
FAK: Focal adhesion kinase
FAO: Fatty acid oxidation
FH: Fumarate hydratase
Fis1: Fission 1
FKBP8: FK506 binding protein 8
FDA: Food and Drug Administration
FIP200: 200KD FAK family kinase-interacting protein
FUNDC1: FUN14 domain containing 1
GLUT4: Glucose transporter 4
GSDMD: Gasdermin D
GSIS: Glucose-stimulated insulin secretion
hAFScs: Human amniotic fluid-derived stem cells
HBV: Hepatitis B virus
HCV: Hepatitis C virus
HD: Huntington’s disease
HIF: Hypoxia-inducible factor
HSP: Heavy strand promoter
HTRA2: High-temperature demand protein A2
Hsp60: The 60 kDa heat shock protein
IDH2: Isocitrate dehydrogenase 2
IFN: Interferon
IL: Interleukin
IMM: Inner mitochondrial membrane
IMS: Intermembrane space
IR: Insulin resistance
ISCs: Intestinal stem cells
JNK: Jun N-terminal kinase
KLF-1: Krüppel-like factor-1
KSS: Kearns-Sayre syndrome
LC3: Light chain 3
LDL: Low-density lipoprotein
LHON: Leber hereditary optic neuropathy
LIR: LC3 interaction region
L-OPA1: Long OPA1
LRP1: Low-density lipoprotein receptor-related protein-1
LRRK2: Leucine-rich repeat kinase 2
LSP: Light strand promoter
MAPK: Mitogen-activated protein kinase
MARCH5: Membrane-associated RING Finger protein 5
MASLD: Metabolic dysfunction-associated steatotic liver disease
MCU: Mitochondrial calcium uniporter
Mdivi-1: Mitochondrial division inhibitor-1
MDV: Mitochondria-derived vesicles
MEF2: The myocyte enhancer factor 2
Mff: Mitochondrial fission factor
MiD: Mitochondrial dynamics protein
Mfn1: Mitofusin 1
Mfn2: Mitofusin 2
MIPS: Mitochondrial information processing system
Miro: Mitochondrial Rho GTPase
Mitophagy: Mitochondrial autophagy
MitoQ: Mitoquinone
mitoRE: Mitochondrial targeted restriction endonuclease
mitoTALEN: Mitochondrial targeted transcriptional activator-like effector nuclease
MLKL: Mixed lineage kinase domain-like
MOTS-c: The mitochondrial open reading frame of 12S rRNA-c
mPTP: Mitochondrial permeability transition pore
MPTP: 1-Methyl-4-phenyl-1,2,5,6-tetrahydropyridine
MSCs: Mesenchymal stem cells
mtDNA: Mitochondrial deoxyribonucleic acid
mTOR: Mechanistic target of rapamycin
mtROS: Mitochondrial reactive oxygen species
MT-TL1: Mitochondrial tRNA leucine 1
mtZFN: Mitochondrial targeted zinc finger nuclease
MT: Mitochondrial transplantation
NAD+: Nicotinamide adenine dinucleotide
NADH: Nicotinamide adenine dinucleotide
NAFLD: Non-alcoholic fatty liver disease
NASH: Non-alcoholic steatohepatitis
NCLX: Na^+^/Ca^2+^ exchanger
ND: NADH-ubiquinone oxidoreductase chain
nDNA: Nuclear DNA
NDP52: Nuclear dot protein 52
NFE2L2: Nuclear factor erythroid 2-like 2
NF-κB: Nuclear factor-κB
NFTs: Neurofibrillary tangles
NIX: Nip3-like protein X
NLRP3: NOD-like receptor protein 3
NMN: Nicotinamide mononucleotide
NO: Nitric oxide
NRF1: Nuclear respiratory factors 1
NRF2: Nuclear respiratory factors 2
NURR1: Nuclear receptor-related 1
OPA1: Optic atrophy protein 1
OPTN: Optineurin
OMM: Outer mitochondrial membrane
oxLDL: Oxidized LDL
OXPHOS: Oxidative phosphorylation
PAMP: Pathogen-associated molecular patterns
PD: Parkinson’s disease
PD-L1: Programmed death-ligand 1
PEP: CSTSMLKAC
PGAM5: Phosphoglycerate mutase 5
PGC-1α: PPARγ coactivator-1alpha
PHB2: Prohibitin 2
PINK1: PTEN induced putative kinase 1
PI3K: Phosphatidylinositol-3-kinase
PKA: cAMP-protein kinase A
POLG: Polymerase gamma
PRC: PPARγ-related coactivator 1
PRKA: Protein kinase cAMP-activated
PRRs: Pattern-recognition receptors
PPARγ: Peroxisome proliferator-activated receptor γ
PTEN: Phosphatase and tensin homolog
RG-PGC-1α: PGC-1α reference gene
RIPK: Receptor-interacting protein kinase
ROS: Reactive oxygen species
RsDdCBE: RsDddA-derived cytosine base editor
RsDddA: DddAtox homolog from Ruminococcus sp. AF17-6
Ub: Ubiquitin
S3: 15-oxospiramilactone
SARS-CoV-2: Severe acute respiratory syndrome coronavirus 2
SDH: Succinate dehydrogenase
SIRT1: Sirtuin 1
SMA: Spinal muscular atrophy
SMAC: Second mitochondrial activator of caspases
SN: Substantia nigra
SNpc: SN pars compacta
SOD2: Superoxide dismutase 2
S-OPA1: Short OPA1
SS: Szeto-Schiller
T2DM: Type 2 diabetes mellitus
TALE: Transcription-activator-like effector
TALEDs: Transcription-activator-like effector -linked deaminases
TAMs: Tumor-associated macrophages
TBK1: TANK-binding kinase 1
TCA: Tricarboxylic acid
TIM: The inner membrane translocase complex
TLR: Toll-like receptor
TME: Tumor microenvironment
TMPRSS2: Transmembrane serine protease 2
TNF-α: Tumor necrosis factor α
TNTs: Tunneling nanotubes
TPP: Triphenylphosphonium
TRAK: Trafficking kinesin-binding protein
UCHL-1: Ubiquitin carboxyl-terminal hydrolase ligase-1
UCP2: Uncoupling protein 2
ULK1: Unc-51-like autophagy activating kinase 1
UPR: Unfolded protein response
USP19: Ubiquitin-specific peptidase 19
VDAC: Voltage-dependent anion channels
VPS13 C: Vacuolar protein sorting 13C
VSMCs: Vascular smooth muscle cells
XIAP: X-linked inhibitor of apoptosis
